# Genetic association between renal function and hearing loss: a bidirectional Mendelian randomization study

**DOI:** 10.1016/j.bjorl.2026.101850

**Published:** 2026-06-19

**Authors:** Jun Wang, Xingxing Chen, Linglin Zhou, Yujie Liu, Sulin Zhang, Haowen Huang

**Affiliations:** a Nanchang University, Jiangxi Medical College, The First Affiliated Hospital, Department of Otorhinolaryngology, Head and Neck Surgery, Nanchang, China; b Otorhinolaryngology Institute of Jiangxi Province, Nanchang, China; c National Clinical Research Center for Otolaryngologic Diseases, Jiangxi Branch Center, Jiangxi, China; d School of Public Health, Wuhan University, Wuhan, China; e Nanchang University, Jiangxi Medical College, The Second Clinical Medical College, Nanchang, China; f Huazhong University of Science and Technology, Tongji Medical College, Union Hospital, Department of Otorhinolaryngology, Wuhan, China; g The First Affiliated Hospital of Nanchang University, Department of Nephrology, Nanchang, China

**Keywords:** Hearing loss, Renal function, Risk, Causality, Mendelian randomization

## Abstract

**Objective:**

Evidence from observational epidemiology studies suggested that renal dysfunction was associated with hearing loss. Whether this represents a causal, reverse causal or correlative relationship remains unclear. We investigated this using a bidirectional two-sample Mendelian Randomization (MR) approach.

**Methods:**

We retrieved genetic variants associated with renal function biomarkers, including creatinine-based eGFR (eGFR-crea), cystatin C-based eGFR (eGFR-cys), urine albumin-to-creatinine ratio (UACR), and serum urate, from the CKDGen Consortium. The summary-level datasets of hearing loss were retrieved from the GWAS of UK Biobank. Inverse-Variance Weighted (IVW) method was the main analysis.

**Results:**

The MR analysis revealed a significant association between genetically predicted UACR and the risk of hearing loss (IVW: OR = 1.038, 95% CI 1.002–1.074, p = 0.036). However, we did not find evidence for a causal effect of eGFR-crea (IVW: OR = 1.046, 95% CI 0.953–1.149, p = 0.342), eGFR-cys (IVW: OR = 0.997, 95% CI 0.940–1.058, p = 0.932) and urate (IVW: OR = 0.999, 95% CI 0.990–1.009, p = 0.904) on hearing loss. Moreover, we found no evidence that hearing loss had causal effects on renal function in the reverse MR analyses. Sensitivity analyses also confirmed the robustness of these findings.

**Conclusion:**

This study provides genetic evidence that elevated UACR is a potential risk factor for hearing loss, but does not support a causal role of hearing loss on kidney function.

**Level of evidence:**

Level 2.

## Highlights

• UACR was a potential risk factor for hearing loss.
• Increased UACR levels significantly raising the risk of hearing loss.
• No causal association was observed between eGFR-crea, eGFR-cys, urate, and hearing loss.

## Introduction

Hearing loss is one of the most frequently occurring sensory disabilities, which affects 6.1% of the world’s population.^1^ It not only leads to communication challenges in daily life but also adversely impacts an individual's cognitive and psychosocial functions.^2^ This condition has the potential to result in social isolation, cognitive decline, financial strain, and a low health-related quality of life.^1^^,^^3^^,^^4^ Given that most instances of hearing loss are acquired and challenging to reverse, yet preventable, investigating the risk factors of hearing loss bears profound significance.

Chronic Kidney Disease (CKD) represents a significant public health challenge, giving rise to a spectrum of multi-systemic complications. Previous studies have suggested there may be a causal association between CKD and hearing loss, with reported frequencies ranging from 36% to 77% in various studies.^5^^,^^6^ Some population-based studies have indicated that a decrease the estimated Glomerular Filtration Rate (eGFR) reduction was associated with hearing loss,^7^, ^8^, ^9^ while another population study in the United States indicated that there was no association between eGFR and hearing loss.^10^ Hence, the causative relationship between renal dysfunction and hearing loss remains contentious. There is a lack of high quality, prospective studies which comprehensively assess the potential association.

Mendelian Randomization (MR) has emerged as a robust genetic research methodology, harnessing genetic variations to surmount certain constraints inherent in observational studies and estimate causal relationships.^11^^,^^12^ MR employs Single Nucleotide Polymorphisms (SNPs) as instrumental variables to gauge the causal effects of an exposure on an outcome.^11^ MR, conceptually akin to a prospective randomized controlled trial, evaluates environmental risk factors and the etiology of illnesses using Mendel's law of independent assortment.^13^ As two-sample MR analysis is especially valuable when exposure and outcome summary statistics come from different studies, enabling researchers to utilize existing datasets without individual-level data.

**Fig. 1:**
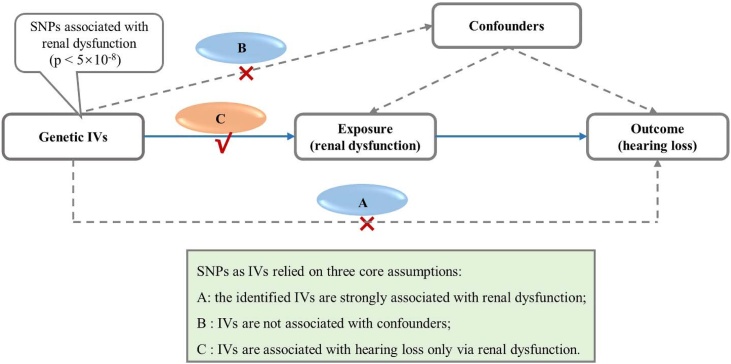
Assumptions and study design flowchart of the MR study. The MR method is based on three core assumptions: (A) The identified IVs are strongly associated with renal dysfunction; (B) IVs are not associated with confounders; (C) IVs are associated with hearing loss only via renal dysfunction. MR, Mendelian Randomization; SNP, Single-Nucleotide Polymorphism; IVs, Instrumental Variables.

In this study, we performed a bidirectional two-sample MR analysis, utilizing summary-level data from the largest GWAS to date on renal function^14^, ^15^, ^16^ and hearing loss (Fig. 1).^17^ The primary aim was to explore the potential causal impact of renal function on hearing loss. Assessments of renal function included estimated GFR based on serum creatinine (eGFR-crea), eGFR based on serum cystatin C (eGFR-cys),^14^ Urine Albumin-to-Creatinine Ratio (UACR),^15^ and serum urate.^16^

## Methods

### Ethics statement

This study adheres to the principles outlined in the Declaration of Helsinki and leverages publicly accessible summary statistics from various GWAS sources.^14^, ^15^, ^16^, ^17^ For this two-sample MR study, no primary data collection was undertaken. Ethical approvals and informed consent details for each participant involved in the constituent studies are available within the original publications.^14^, ^15^, ^16^, ^17^

### Data sources for renal function

For the exposure data set of renal functions, the summary-level GWAS data correlated with kidney function that were obtained from a meta-analysis of the GWAS of the Chronic Kidney Disease Genetics (CKDGen) Consortium. The meta-analysis of GWAS data for eGFR-crea (n = 1,004,040), eGFR-cys (n = 460,826), UACR (n = 547,361) and urate (n = 288,649) among individuals of European ancestry.^14^, ^15^, ^16^ The study characteristics of the four GWAS meta-analyses are detailed in Supplementary Table S1.

### Data sources for hearing loss

As for the outcome datasets of the GWAS, data on hearing loss was obtained from a GWAS using a UK Biobank sample of European descent (n = 250,389), the largest and latest hearing difficulty GWAS data.^17^ The study characteristics of the GWAS meta-analyses are detailed in Supplementary Table S1.

### Selection of genetic instruments

We selected the genetic instruments for this study based on published criteria.^18^^,^^19^ The selection criteria were as follows: (i) SNPs significantly associated with the exposures (p < 5 × 10^−8^); (ii) Linkage Disequilibrium (LD) r^2^ < 0.001 and clump window <10,000 kb. Furthermore, palindromic genetic variants were removed during the harmonization of the genetic variants. We then utilized F-statistics to evaluate the strength of the instruments. We included genetic variants with sufficient strength and considered F-statistics >10 as sufficient strength.^20^^,^^21^ Additionally, to rule out the influence of known confounders on the causality assessment, potential secondary phenotypes of the selected SNPs were manually browsed with the PhenoScanner (http://www.phenoscanner.medschl.cam.ac.uk), a platform with comprehensive information on the association of genotype and phenotype, to see whether these SNPs were associated with the potential risk factors. Lastly, Due to the potential violation of certain eGFR loci by creatine metabolism, we turned to the GWAS dataset of Blood Urea Nitrogen (BUN) from the CKDGen Consortium. This alternative marker of renal dysfunction was utilized to eliminate SNPs that were less likely to be associated with renal dysfunction.^22^ Finally, SNPs inverse, significantly associated with BUN (p < 0.05) were selected as genetic instruments for eGFR.

### MR analyses

We conducted a two-sample bidirectional MR analysis to evaluate the causal relationships between renal function and hearing loss. Four different methods of MR [random-effect Inverse-Variance Weighted (IVW), MR Egger, weighted median, and maximum likelihood] were performed to address variant heterogeneity and the pleiotropy effect. IVW stands as the predominant method utilized in MR analysis, as it amalgamates the Wald ratio estimated for each SNP on the outcome, culminating in a consolidated causal estimate. Moreover, three supplementary MR analyses were undertaken alongside IVW, aiming to furnish more resilient estimates across a diverse spectrum of scenarios.

The subsequent techniques were employed to execute sensitivity analyses. Firstly, the Cochran’s *Q* test was applied to identify potential heterogeneity in estimated values based on IVW of SNPs (the presence of heterogeneity was inferred at p < 0.05). Secondly, we employed the MR-Egger intercept method to assess whether any evidence of horizontal pleiotropy existed among the selected SNPs (the presence of horizontal pleiotropy was inferred at p < 0.05). Thirdly, we implemented the MR Pleiotropy RESidual Sum and Outlier (MR-PRESSO) approach to identify and subsequently exclude any outliers, thus refining the estimates. Furthermore, we adopted a leave-one-out analysis to scrutinize whether any individual SNP significantly influenced the outcome. Lastly, we utilized a funnel plot to directly assess the presence of pleiotropy. Additionally, a power analysis ）was conducted to estimate the minimum effect size that could be detected per exposure (Supplementary Table S8).

MR estimates are presented as Odds Ratios (ORs) with corresponding 95% Confidence Intervals (95% CIs). A two-tailed p-value of less than 0.05 was considered statistically significant. All MR analyses and data management were performed by using R software package (version 4.3.1, the R Foundation for Statistical Computing, Vienna, Austria).

## Results

### Genetic variants selection

**Fig. 2:**
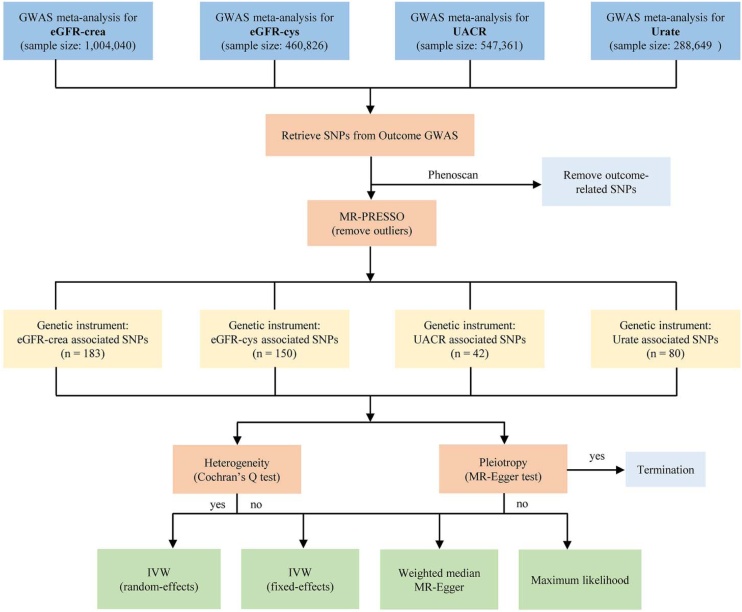
Flow chart of the Mendelian randomization analysis. MR, Mendelian Randomization; SNP, Single‐Nucleotide Polymorphism; IV, Instrumental Variable; GWAS, Genome-Wide Association Study; eGFR-crea, estimated Glomerular Filtration Rate based on creatinine; eGFR-cys, estimated Glomerular Filtration Rate based on serum cystatin C; UACR, Urine Albumin-to-Creatinine Ratio; IVW, Inverse-Variance Weighted.

We derived Instrumental Variables (IVs) that were significantly associated with renal dysfunction from the largest to data GWAS. After a comprehensive investigation utilizing the PhenoScanner database, certain SNPs were removed due to their links with potential confounding factors, including body mass index, obesity, hypertension, diabetes, and hyperlipemia (Supplementary Table S2). Additionally, we excluded palindromic SNPs (A/T or G/C) and SNPs that were not available in relation to the desired outcome. Finally, we removed potential outliers by using the MR-PRESSO test and examined sensitivity plots to select our genetic variants in Supplementary Table S3. Importantly, the *F*-statistics for these selected genetic instruments surpassed the conventional threshold of 10 (Supplementary Tables S4‒S7), indicating the robustness of our instruments.^23^ In total, 183 genome-wide significant index SNPs were associated with eGFR-crea, 150 index SNPs were used to genetically predict eGFR-cys, 42 index SNPs were selected to genetically predict UACR and 80 SNPs predict urate (Fig. 2).

### MR and sensitivity analyses

**Fig. 3:**
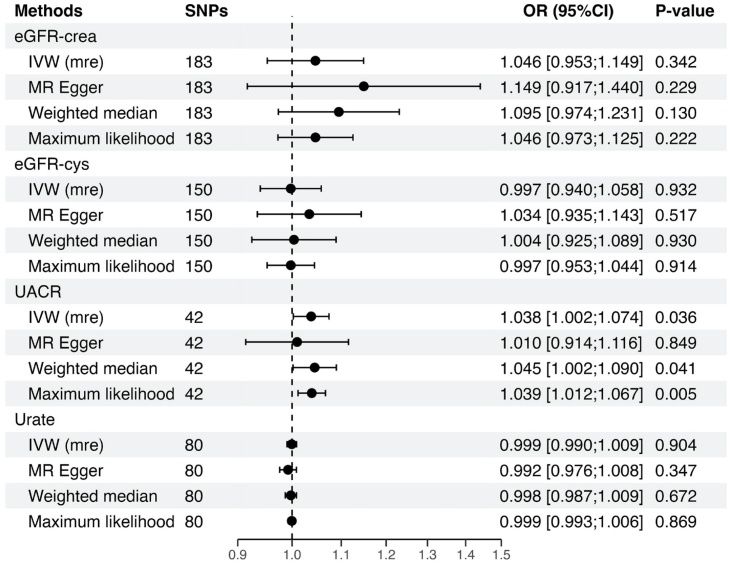
Forest plot which visualizes the MR analyses between renal function and hearing loss. OR represent a genetically determined 1 unit increase of ln(eGFR-crea), 1 unit increase of ln(eGFR-cys), 1 unit increase of ln(UACR), and 1 unit increase in the odds of urate, respectively (renal dysfunction as exposure) with the odds of hearing loss. Alternatively, the OR represent a genetically determined 1 unit increase in the odds of hearing loss. eGFR-crea, estimated Glomerular Filtration Rate based on creatinine; eGFR-cys, estimated Glomerular Filtration Rate based on serum cystatin C; UACR, Urine Albumin-to-Creatinine Ratio; IVW, Inverse-Variance Weighted, MR, Mendelian Randomization; SNPs, Single-Nucleotide Polymorphisms; OR, Odds Ratio; CI, Confidence Interval.

**Fig. 4:**
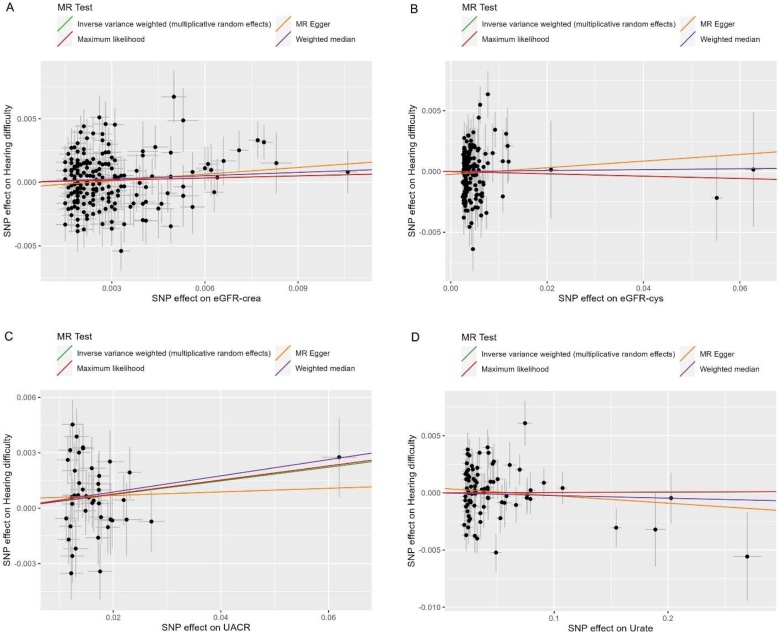
Scatter plot of genetic correlations of renal function biomarkers and hearing loss using different MR methods. (A) The effect size and 95% CI of each SNP on eGFR-crea and hearing loss risk. (B) The effect size and 95% CI of each SNP on eGFR-cys and hearing loss risk. (C) The effect size and 95% CI of each SNP on UACR and hearing loss risk. (D) The effect size and 95% CI of each SNP on urate and hearing loss risk. X-axis represents genetic effect of each SNP on eGFR-crea (A), eGFR-cys (B), UACR (C) or urate (D). Y-axis reflects the genetic effect of each SNP on hearing loss risk. eGFR-crea, estimated Glomerular Filtration Rate based on creatinine; eGFR-cys, estimated Glomerular Filtration Rate based on serum cystatin C; UACR, Urine Albumin-to-Creatinine Ratio; MR, Mendelian Randomization; SNPs, Single-Nucleotide Polymorphisms.

**Fig. 5:**
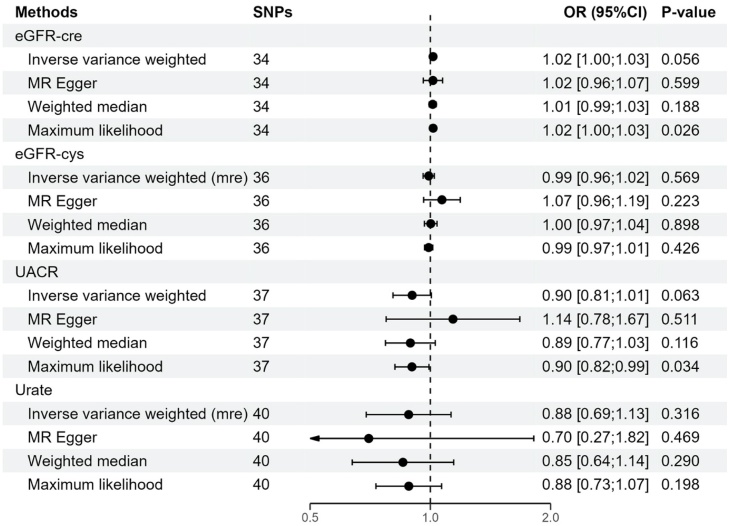
Forest plots illustrated the causal associations between hearing loss and renal function using various methods. eGFR-crea, estimated Glomerular Filtration Rate based on creatinine; eGFR-cys, estimated Glomerular Filtration Rate based on serum cystatin C; UACR, Urine Albumin-to-Creatinine Ratio; IVW, Inverse-Variance Weighted, MR, Mendelian Randomization; SNPs, Single-Nucleotide Polymorphisms; OR, Odds Ratio; CI, Confidence Interval.

The main MR analysis based on the IVW method supported a causal effect of UACR on hearing loss risk (n = 42 SNPs, OR = 1.038, per 1 unit increase in the odds for UACR, 95% CI: 1.002–1.074, p = 0.036) (Figs. 3 and 4 ). Our additionally analysis based on the MR-Egger, weighted median, and maximum likelihood were in general concordant with the results of the IVW method. More specifically, the weighted median analysis also supported a causal effect of UACR on hearing loss risk (n = 42 SNPs, OR = 1.045, per 1 unit increase in the odds for UACR, 95% CI: 1.002–1.090, p = 0.041) (Figs. 3 and 4). The point estimates of the MR-Egger slope method were also in general in line with the point estimates of the IVW method. However, we did not find evidence for a causal effect of eGFR-crea (IVW: OR = 1.046, 95% CI 0.953–1.149, p = 0.342), eGFR-cys (IVW: OR = 0.997, 95% CI 0.940–1.058, p = 0.932) and urate (IVW: OR = 0.999, 95% CI 0.990–1.009, p = 0.904) on hearing loss (Fig. 3). The effect estimates of the genetic variants associated with eGFR-crea, eGFR-cys, UACR, and urate that were used in our two-sample MR analyses are extensively presented in Supplementary Table S8. Additionally, to investigate if hearing loss causally affects specific renal function, we also conducted a reverse MR analysis (Supplementary Tables S9‒S12). Our results indicated that hearing loss was not a causal factor for renal function (Fig. 5).

In sensitivity analyses, we employed a range of methods, including MR-Egger intercept, leave-one-out test, and funnel plot, for both statistically significant and nominally significant estimates. Although the results exhibit a certain degree of heterogeneity, they are free from horizontal pleiotropy, which to some extent proves that the results are robust (Table 1). In addition, leave-one-out analyses and funnel plots are shown in Supplementary Figure S1 and S2. The estimates were not biased by single SNP, indicating that estimates were not violated.

**Table 1:** Associations of genetically predicted renal dysfunction biomarkers with risk of hearing loss by sensitivity analysis.

Exposure	Outcome	Heterogeneity test MR-Egger			Heterogeneity test IVW			Pleiotropy test		
		Q	Q_df	Q_p-value	Q	Q_df	Q_p-value	Egger intercept	SE	p-value
eGFR-crea	Hearing loss	303.87	181	2.86E-08	305.22	182	2.83E-08	−3.055E-04	3.417E-04	0.372
eGFR-cys	Hearing loss	250.39	148	2.96E-07	251.66	149	2.97E-07	−2.297E-04	2.657E-04	0.389
UACR	Hearing loss	71.32	40	1.68E-03	71.91	41	2.02E-03	4.921E-04	8.599E-04	0.570
Urate	Hearing loss	147.49	78	3.40E-06	149.64	79	2.79E-06	4.262E-04	3.997E-04	0.290
Hearing loss	eGFR-crea	43.41	32	0.09	43.41	33	0.11	5.24E-06	2.09E-04	0.980
Hearing loss	eGFR-cys	63.41	34	1.6E-03	67.53	35	7.6E-4	−5.90E-04	3.96E-04	0.147
Hearing loss	UACR	44.99	35	0.12	47.00	36	0.10	−1.78E-03	1.43E-03	0.220
Hearing loss	Urate	67.03	38	2.5E-03	67.45	39	3.13E-03	1.69E-03	3.44E-03	0.627

MR, Mendelian Randomization; IVW, Inverse-Variance Weighted; *Q*, heterogeneity statistic Q; and Q_df, Q degree of freedom; SE, Standard Error. Note: Three SNPs (rs1214761, rs693906, rs753381) that were removed due to potential horizontal pleiotropy (eGFR-crea as exposure); No SNP was excluded (eGFR-cys and UACR as exposure); and one SNPs (rs8050136) that was removed due to potential horizontal pleiotropy (Urate as exposure).

## Discussion

We evaluated the causal relationship between kidney function and the risk of hearing loss based on the use of large available GWAS statistics. This study revealed that the genetically predicted kidney function, especially increased UACR, may as an independent risk factor of hearing loss. However, we did not find evidence to support the causality of hearing loss on kidney function. This has important implications for research efforts that attempt to identify prevention and treatment targets for both CKD and hearing loss.

A close association between CKD and hearing loss has been repeatedly reported by observational studies. For example, the Blue Mountains Hearing Study (n = 2,564^8^ presented the first community-based study on an Australian population and demonstrated an association between renal dysfunction and hearing loss. Another cross-sectional study of 12,508 individuals from the Chinese Health and Retirement Longitudinal Study indicated that kidney dysfunction, such as lower eGFR, was independently associated with hearing loss.^7^ Furthermore, the Korean National Health and Nutrition Examination Survey (n = 10,608^24^ also found that individuals with low-grade UACR are more likely to have hearing impairment. However, the Epidemiology of Hearing Loss Study (n = 15,676^10^ showed that there is no significant association between eGFR-crea and risk of incident hearing loss. The validity of these observational findings could potentially be compromised by unaccounted confounding variables and the possibility of reverse causation. These factors erode the reliability of the outcomes. Therefore, it's imperative to establish the presence of a causal relationship between the two conditions.

Although the precise mechanism linking renal function with hearing loss has not been fully elucidated, several possible rationales have been proposed. One potential mechanism could arise from the mutual antigenicity and analogous physiological mechanisms shared between the kidney and cochlea. These mechanisms encompass the transportation of fluid and electrolytes, possibly elucidating the connection between kidney disease and hearing loss.^25^ The cochlea's stria vascularis and the kidney's glomerulus are epithelial structures that share close ties with the vascular system.^26^ Numerous ion channels and transporters responsible for the cycling of K^+^ and the maintenance of endolymphatic K^+^, Na^+^, Ca^2+^, and pH balance are expressed in both the inner ear and the kidney.^25^^,^^26^ Additionally, in experimental uremic guinea pigs, a notable reduction in Na^+^-K^+^-ATPase activity within the inner ear has been observed. Given the enzyme's essential role in upholding the electrochemical gradient in the ear, its inhibition might constitute a primary factor in hearing impairment.^27^ Furthermore, potential connections emerge from kidney dysfunction's common association with microvascular damage and endothelial dysfunction. These factors could potentially induce hearing loss by disrupting the cochlear microcirculation.^28^

In this study, we further utilized two-sample MR analysis to determine the causal effects between renal function and hearing loss. Our results confirm that renal dysfunction, in particular increased UACR, is an independent risk factor for hearing loss. However, the causal effect estimates derived from our MR analysis diverged from the effect estimates reported in earlier observational epidemiological investigations.^7^, ^8^, ^9^, ^10^ We did not find evidence for a causal role of eGFR-crea, eGFR-cys and urate on hearing loss, although the effect estimates of these assessments were in line with each other. This disparity might stem from variations in the temporal scope between MR studies and conventional observational inquiries. While MR studies compute risk estimates based on lifelong exposure to specific risk factors, traditional observational studies gauge risk estimates with a designated follow-up period.^19^ The MR analysis serves as a valuable and enlightening research methodology capable of overcoming latent confounding influences and reversing causation concerns.^11^

Our MR study confirmed that the genetically predicted kidney dysfunction, especially increased UACR, is an independent risk factor of hearing loss. This could be due to the reason that the accumulation or excretion of serum creatinine and cystatin C lagging behind changes in renal function,^29^ both eGFR-crea and eGFR-cys are unable to accurately reflect the progression of renal damage or the recovery of renal function compared to UACR.^30^ Furthermore, kidneys possess robust compensatory capabilities, wherein during the initial phases of CKD, eGFR might not exhibit a decline; in some cases, it might even show a marginal increase.^31^ Additionally, UACR is often considered a marker of microvascular damage and filtration barrier.^32^^,^^33^ Elevated UACR result in a substantial loss of albumin in the body, causing a prominent reduction in colloid osmotic pressure within the blood vessels. Consequently, it leads to a decrease in plasma volume within the vessels, accompanied by a notable increase in interstitial tissue fluid and lymphatic content. Situated on the lateral wall of the cochlea, the vascular stria vascularis is a multi-layered, epithelial cell layer with abundant vascularity that borders the endolymph on its apical surface.^34^ The integrity of hearing function can be compromised by even subtle vascular alterations, given that the inner ear, especially the stria vascularis, heavily relies on undisturbed local blood circulation.^35^^,^^36^ Therefore, we speculate that significant protein loss in the kidneys can lead to a considerable elevation of extralymphatic fluid in the cochlea, exerting pressure on the vascular stria and subsequently causing damage to the hair cells and spiral ganglion. These subtle vascular changes might result in decreased oxygen supply to the cochlea, ultimately culminating in hearing impairment.^37^^,^^38^

Our study has several strengths and limitations. Firstly, this study is the first to explore the causal link between renal function and hearing loss using a two-sample MR analysis, avoiding confounders and reverse causation common in observational studies. In addition, multiple sensitivity analyses, and IV strength evaluations, were conducted to ensure the robustness and effectiveness of the results. There are also some limitations. First, the MR analysis is based on specific assumptions that cannot be assessed. Secondly, the present study was mainly conducted on individuals of European ancestry, which limits the generality of our results to other populations. Thirdly, we use SNPs associated with renal function as IVs to explore causality, but it is impossible to infer the causality between the kidney function and the severity of hearing loss. Lastly, despite our genetic-centric exploration of the connection between renal function and hearing loss, the underlying mechanisms remain obscure and require further investigation.

## Conclusions

The present study provides genetic support that higher UACR levels is a potential risk factor for hearing loss. In addition, the evidence did not support a significant causal association of hearing loss on kidney function. These findings provide novel perspectives for exploring shared pathological mechanisms between CKD and hearing impairments. Despite this, intervention studies with higher level of evidence are still needed to finally clarify the subject.

## ORCID ID

Xingxing Chen: 0000-0002-0548-7116

Linglin Zhou: 0009-0000-2292-7657

Yujie Liu: 0009-0008-8803-9597

Sulin Zhang: 0000-0002-3609-6804

Haowen Huang: 0000-0002-3882-2291

## Authors' contributions

JW: Study conception and design; Drafting the article and figures; Writing and editing. HWH: Data acquisition and analysis; Writing and editing. XXC: Software; Editing. LLZ & YJL: Review and editing. HWH & SLZ: Conceptualization; Project administration; Review and editing. All co-authors contributed to the article and approved the submitted version.

## Funding

This work was supported by the Young Scientists Fund of the National Natural Science Foundation of China (grant nº 82501420); Jiangxi Provincial Natural Science Foundation Youth Project (grant nº 20252BAC200112), and the First Affiliated Hospital of Nanchang University Clinical Research and Cultivation Project (grant nº YFYLCYJPY202437).

## Data availability statement

The authors declare that all data are available in repository.

## Availability of data

The original contributions presented in the study are included in the article/Supplementary Material. All data are publicly available. The summary statistics from GWAS meta-analyses on renal dysfunction are available at URL: https://ckdgen.imbi.uni-freiburg.de/. The summary statistics from the GWAS meta-analysis on hearing loss are available at URL: https://zenodo.org/record/3490750#.YvZrAdNBwb5.

## Declaration of competing interest

The authors declare no conflicts of interest.

## Footnotes

Appendix A Supplementary material related to this article can be found, in the online version, at doi:https://doi.org/10.1016/j.bjorl.2026.101850.

## References

[1] Olusanya B.O., Davis A.C., Hoffman H.J. (2019). Hearing loss grades and the International classification of functioning, disability and health. Bull World Health Organ..

[2] Wang J., Chen X.X., Liu D. (2023). Association of hearing status and cognition with fall among the oldest-old Chinese: a nationally representative cohort study. Ear Hear..

[3] Rutherford B.R., Brewster K., Golub J.S., Kim A.H., Roose S.P. (2018). Sensation and psychiatry: linking age-related hearing loss to late-life depression and cognitive decline. Am J Psychiatry.

[4] Bainbridge K.E., Wallhagen M.I. (2014). Hearing loss in an aging American population: extent, impact, and management. Annu Rev Public Health..

[5] Agrawal M., Singh C.V. (2023). Sensorineural hearing loss in patients with chronic kidney disease: a comprehensive review. Cureus..

[6] Sarin V., Sharma A., Chopra I. (2022). High frequency hearing loss in chronic renal disease: a cross-sectional study. Indian J Otolaryngol Head Neck Surg..

[7] Liu W., Meng Q., Wang Y. (2020). The association between reduced kidney function and hearing loss: a cross-sectional study. BMC Nephrol..

[8] Vilayur E., Gopinath B., Harris D.C., Burlutsky G., McMahon C.M., Mitchell P. (2010). The association between reduced GFR and hearing loss: a cross-sectional population-based study. Am J Kidney Dis..

[9] Schubert C.R., Paulsen A.J., Nondahl D.M. (2018). Association between cystatin C and 20-year cumulative incidence of hearing impairment in the epidemiology of hearing loss study. JAMA Otolaryngol Head Neck Surg..

[10] Gupta S., Curhan S.G., Cruickshanks K.J., Klein B.E.K., Klein R., Curhan G.C. (2020). Chronic kidney disease and the risk of incident hearing loss. Laryngoscope..

[11] Sekula P., Del Greco M.F., Pattaro C., Köttgen A. (2016). Mendelian randomization as an approach to assess causality using observational data. J Am Soc Nephrol..

[12] Birney E. (2022). Mendelian randomization. Cold Spring Harb Perspect Med..

[13] Davey Smith G., Holmes M.V., Davies N.M., Ebrahim S. (2020). Mendel’s laws, Mendelian randomization and causal inference in observational data: substantive and nomenclatural issues. Eur J Epidemiol..

[14] Stanzick K.J., Li Y., Schlosser P. (2021). Discovery and prioritization of variants and genes for kidney function in >1.2 million individuals. Nat Commun..

[15] Teumer A., Li Y., Ghasemi S. (2019). Genome-wide association meta-analyses and fine-mapping elucidate pathways influencing albuminuria. Nat Commun..

[16] Tin A., Marten J., Halperin Kuhns V.L. (2019). Target genes, variants, tissues and transcriptional pathways influencing human serum urate levels. Nat Genet..

[17] Wells H.R.R., Freidin M.B., Zainul Abidin F.N. (2019). GWAS identifies 44 independent associated genomic loci for self-reported adult hearing difficulty in UK Biobank. Am J Hum Genet..

[18] Wang J., Liu D., Tian E. (2022). Is hearing impairment causally associated with falls? Evidence from a two-sample Mendelian randomization study. Front Neurol..

[19] Geurts S., van der Burgh A.C., Bos M.M. (2022). Disentangling the association between kidney function and atrial fibrillation: a bidirectional Mendelian randomization study. Int J Cardiol..

[20] Hu C., Li Y., Qian Y., Wu Z., Hu B., Peng Z. (2023). Kidney function and cardiovascular diseases: a large-scale observational and Mendelian randomization study. Front Immunol..

[21] Huang H., Ren Y., Wang J. (2024). Renal function and risk of dementia: a Mendelian randomization study. Ren Fail..

[22] Chen X., Kong J., Pan J. (2021). Kidney damage causally affects the brain cortical structure: a Mendelian randomization study. EBioMedicine..

[23] Pierce B.L., Ahsan H., Vanderweele T.J. (2011). Power and instrument strength requirements for Mendelian randomization studies using multiple genetic variants. Int J Epidemiol..

[24] Kang S.H., Jung D.J., Choi E.W. (2015). Association between low-grade albuminuria and hearing impairment in a non-diabetic Korean population: The Korea National Health and Nutrition Examination Survey (2011-2013). Ann Med..

[25] Thodi C., Thodis E., Danielides V., Pasadakis P., Vargemezis V. (2006). Hearing in renal failure. Nephrol Dial Transplant..

[26] Quick C.A., Fish A., Brown C. (1973). The relationship between cochlea and kidney. Laryngoscope..

[27] Adler D., Fiehn W., Ritz E. (1980). Inhibition of Na+,K+-stimulated ATPase in the cochlea of the guinea pig. A potential cause of disturbed inner ear function in terminal renal failure. Acta Otolaryngol.

[28] Cuna V., Battaglino G., Capelli I. (2015). Hypoacusia and chronic renal dysfunction: new etiopathogenetic prospective. Ther Apher Dial..

[29] Molitoris B.A., Reilly E.S. (2016). Quantifying glomerular filtration rates in acute kidney injury: a requirement for translational success. Semin Nephrol..

[30] Provenzano M., Rotundo S., Chiodini P. (2020). Contribution of predictive and prognostic biomarkers to clinical research on chronic kidney disease. Int J Mol Sci..

[31] Melamed M.L., Bauer C., Hostetter T.H. (2008). eGFR: is it ready for early identification of CKD?. Clin J Am Soc Nephrol..

[32] Georgakis M.K., Chatzopoulou D., Tsivgoulis G., Petridou E.T. (2018). Albuminuria and cerebral small vessel disease: a systematic review and meta-analysis. J Am Geriatr Soc..

[33] Zhao S., Yu S., Chi C. (2019). Association between macro- and microvascular damage and the triglyceride glucose index in community-dwelling elderly individuals: the Northern Shanghai Study. Cardiovasc Diabetol..

[34] Suzuki T., Oyamada M., Takamatsu T. (2001). Different regulation of connexin26 and ZO-1 in cochleas of developing rats and of guinea pigs with endolymphatic hydrops. J Histochem Cytochem..

[35] Thulasiram M.R., Ogier J.M., Dabdoub A. (2022). Hearing function, degeneration, and disease: spotlight on the stria vascularis. Front Cell Dev Biol..

[36] Johns J.D., Adadey S.M., Hoa M. (2023). The role of the stria vascularis in neglected otologic disease. Hear Res..

[37] Yamasoba T., Lin F.R., Someya S., Kashio A., Sakamoto T., Kondo K. (2013). Current concepts in age-related hearing loss: epidemiology and mechanistic pathways. Hear Res..

[38] Simões J.F.C.P.M., Vlaminck S., Seiça R.M.F., Acke F., Miguéis A.C.E. (2023). Cardiovascular Risk and Sudden Sensorineural Hearing Loss: A Systematic Review and Meta-Analysis. Laryngoscope..

