# Supplementary material for: Genetic association between renal function and hearing loss: a bidirectional Mendelian randomization study
Source: Braz J Otorhinolaryngol. 2026 Jun 19;92(5):101850. doi: 10.1016/j.bjorl.2026.101850 (PMC13314737; doi:10.1016/j.bjorl.2026.101850)
Supplement: Supplementary file 1 [file mmc1.docx]

**BJORL-D-25-00201**

**Supplementary Tables**

**Table S1** Source and sample size of GWAS summary statistics.

**Table S2** Excluding SNPs with confounding traits at a genome-wide significance level (P < 5E-08).

**Table S3** MR-PRESSO testing casual effect of kidney function on hearing loss.

**Table S4** Effect estimates for the associations of the genetic variants with eGFR_cre and hearing loss.

**Table S5** Effect estimates for the associations of the genetic variants with eGFR_cys and hearing loss.

**Table S6** Effect estimates for the associations of the genetic variants with UACR and hearing loss.

**Table S7** Effect estimates for the associations of the genetic variants with urate and hearing loss.

**Table S8** Power of the models and F-statistics of the instrumental SNPs.

**Table S9** Effect estimates for the associations of the genetic variants with hearing loss and eGFR_cre.

**Table S10** Effect estimates for the associations of the genetic variants with hearing loss and eGFR_cys.

**Table S11** Effect estimates for the associations of the genetic variants with hearing loss and UACR.

**Table S12** Effect estimates for the associations of the genetic variants with hearing loss and urate.

**Table S1** Source and sample size of GWAS summary statistics.

| **Exposures** | **Phenotype** | **Data source** | **Sample size** |
| --- | --- | --- | --- |
|  | eGFR_crea | Stanzick et al. 2021 publication (https://ckdgen.imbi.uni-freiburg.de/) | 1,004,040 |
|  | eGFR_cys | Stanzick et al. 2021 publication (https://ckdgen.imbi.uni-freiburg.de/) | 460,826 |
|  | UACR | Teumer et al. 2019 publication (https://ckdgen.imbi.uni-freiburg.de/) | 547,361 |
|  | Urate | Tin et al. 2019 publication (https://ckdgen.imbi.uni-freiburg.de/) | 288,649 |
| **Outcome** | Hearing difficulty | Wells HRR. et al. 2019 publication (https://zenodo.org/record/3490750#.YvZrAdNBwb5) | 250,389 |

eGFR-crea, estimated Glomerular Filtration Rate based on creatinine; eGFR-cys, estimated Glomerular Filtration Rate based on serum cystatin-C; UACR, Urine Albumin-to-Creatinine Ratio.

**Table S2** Excluding SNPs with confounding traits at a genome-wide significance level (p < 5E-08).

| **Phenoscanner traits associated with the eGFR_cre instrumental SNPs (p < 5E-8)** | | | |
| --- | --- | --- | --- |
| **SNP** | **effect_allele** | **other_allele** | **Confounding traits** |
| rs10224002 | A | G | Chronic kidney disease; Self-reported hypertension; Vascular or heart problems diagnosed by doctor |
| rs223308 | A | G | Number of self-reported non-cancer illnesses; Self-reported hypertension |
| rs35320690 | T | C | Body mass index |
| rs429358 | T | C | Coronary artery disease; Type II diabetes |
| rs4434960 | A | T | Body mass index; Vascular or heart problems diagnosed by doctor: high blood pressure |
| rs4946932 | A | C | Body mass index |
| rs77924615 | A | G | Self-reported hypertension; Vascular or heart problems diagnosed by doctor: high blood pressure |
| rs9932625 | A | G | Self-reported hypertension; Vascular or heart problems diagnosed by doctor: high blood pressure |
| **Phenoscanner traits associated with the eGFR_cys instrumental SNPs (p < 5E-8)** | | | |
| rs10010367 | A | C | Self-reported hypertension; Vascular or heart problems diagnosed by doctor: high blood pressure |
| rs1260326 | T | C | C reactive protein levels or triglyceride levels pleiotropy |
| rs2464196 | A | G | Coronary artery disease; Low density lipoprotein; |
| rs2638281 | A | G | Crohns disease; Self-reported high cholesterol |
| rs3184504 | T | C | Diabetes mellitus type 1; Coronary artery disease |
| rs429358 | T | C | Type II diabetes; Coronary artery disease |
| rs56144347 | T | C | Body mass index; Coronary artery disease |
| rs7206629 | T | C | Body mass index; Type II diabetes |
| rs76798800 | T | G | Body mass index |
| rs7684939 | A | G | Body mass index |
| rs77924615 | A | G | Self-reported hypertension; Vascular or heart problems diagnosed by doctor: high blood pressure |
| **Phenoscanner traits associated with the Urate instrumental SNPs (p < 5E-8)** | | | |
| rs1260326 | T | C | C reactive protein levels or triglyceride levels pleiotropy; Triglycerides |
| rs35198068 | T | C | Body mass index; Type II diabetes; |

**Table S3** MR-PRESSO testing causal effect of kidney function on hearing loss.

| **Exposure: eGFR_cre p < 5E-8 (n = 186)** | | | | | | | | |
| --- | --- | --- | --- | --- | --- | --- | --- | --- |
| **MR Analysis** | **Causal Estimate** | **SD** | **T-stat** | **p-value** | **Global test RSSobs** | **Global test p-value** | **Distortion coefficient** | **Distortion test p-value** |
| Raw | 0.038 | 0.052 | 0.728 | 0.468 | 373.837 | <1e-04 | -16.873 | 0.898 |
| Outlier-corrected | 0.045 | 0.048 | 0.950 | 0.344 | 373.837 | <1e-04 | -16.873 | 0.898 |
| **Exposure: eGFR_cys p < 5E-8 (n = 150)** | | | | | | | | |
| **MR Analysis** | **Causal Estimate** | **SD** | **T-stat** | **p-value** | **Global test RSSobs** | **Global test p-value** | **Distortion coefficient** | **Distortion test p-value** |
| Raw | -0.003 | 0.030 | -0.085 | 0.932 | 254.909 | <1e-04 | NA | NA |
| Outlier-corrected | NA | NA | NA | NA | 254.909 | <1e-04 | NA | NA |
| **Exposure: UACR p < 5E-8 (n = 42)** | | | | | | | | |
| **MR Analysis** | **Causal Estimate** | **Sd** | **T-stat** | **p-value** | **Global test rssobs** | **Global test p-value** | **Distortion coefficient** | **Distortion test p-value** |
| Raw | 0.037 | 0.018 | 2.094 | 0.425 | 74.610 | 0.002 | NA | NA |
| Outlier-corrected | NA | NA | NA | NA | 74.610 | 0.002 | NA | NA |
| **Exposure: Urate p < 5E-8 (n = 81)** | | | | | | | | |
| **MR Analysis** | **Causal Estimate** | **Sd** | **T-stat** | **p-value** | **Global test rssobs** | **Global test p-value** | **Distortion coefficient** | **Distortion test p-value** |
| Raw | -0.002 | 0.005 | -0.358 | 0.722 | 190.040 | <1e-04 | -228.399 | 0.157 |
| Outlier-corrected | -0.001 | 0.005 | -0.121 | 0.904 | 190.040 | <1e-04 | -228.399 | 0.157 |

**Table S4** Effect estimates for the associations of the genetic variants with eGFR_cre and hearing loss.

| **SNP** | **effect_allele** | **other_allele** | **beta.exposure** | **beta.outcome** | **eaf.exposure** | **eaf.outcome** | **se.exposure** | **pval.exposure** | **pval.outcome** | **F_statisic** |
| --- | --- | --- | --- | --- | --- | --- | --- | --- | --- | --- |
| rs10151563 | A | G | -0.0019 | 0.0015029 | 0.6048 | 0.607094 | 3.00E-04 | 1.16E-11 | 0.27 | 19.1748 |
| rs10224002 | A | G | 0.0077 | 0.0033118 | 0.7152 | 0.712609 | 3.00E-04 | 3.55E-142 | 0.023 | 268.44274 |
| rs10430743 | T | G | 0.0027 | -0.0005045 | 0.4278 | 0.42604 | 3.00E-04 | 1.68E-22 | 0.71 | 39.657007 |
| rs10846156 | T | G | -0.004 | 0.0029742 | 0.8037 | 0.798035 | 3.00E-04 | 4.13E-31 | 0.071 | 56.09771 |
| rs10851885 | A | G | 0.0056 | 0.0008153 | 0.7514 | 0.742278 | 3.00E-04 | 9.74E-69 | 0.59 | 130.1941 |
| rs10862375 | C | G | -0.0019 | 0.0007448 | 0.2762 | 0.283387 | 3.00E-04 | 9.82E-10 | 0.61 | 16.037734 |
| rs1087289 | T | G | 0.0019 | -5.401E-05 | 0.6103 | 0.612807 | 3.00E-04 | 3.23E-10 | 0.97 | 19.079889 |
| rs10994860 | T | C | 0.0044 | 0.002772 | 0.1844 | 0.178078 | 4.00E-04 | 3.50E-36 | 0.11 | 36.397234 |
| rs11062102 | T | C | 0.0041 | -0.0003776 | 0.3397 | 0.33704 | 3.00E-04 | 2.32E-47 | 0.79 | 83.796798 |
| rs11071738 | T | C | -0.0026 | -0.0015433 | 0.5276 | 0.52131 | 3.00E-04 | 5.97E-21 | 0.24 | 37.442444 |
| rs11109717 | T | C | -0.0017 | 0.0020244 | 0.6973 | 0.693396 | 3.00E-04 | 8.40E-09 | 0.16 | 13.555715 |
| rs111541038 | T | C | 0.0032 | -0.0010432 | 0.8591 | 0.857876 | 4.00E-04 | 2.60E-14 | 0.58 | 15.494249 |
| rs112297695 | T | C | 0.0018 | 0.000464 | 0.3354 | 0.320411 | 3.00E-04 | 1.30E-09 | 0.74 | 16.049517 |
| rs1126393 | T | C | -0.0018 | -0.0027504 | 0.626 | 0.619566 | 3.00E-04 | 9.54E-11 | 0.043 | 16.857177 |
| rs1133415 | A | G | 0.0022 | -0.0008924 | 0.4758 | 0.472062 | 3.00E-04 | 3.57E-16 | 0.5 | 26.826563 |
| rs113441031 | T | C | -0.003 | 0.003109 | 0.1651 | 0.172078 | 4.00E-04 | 1.29E-16 | 0.077 | 15.507432 |
| rs113572081 | C | G | -0.0028 | -0.0023552 | 0.1369 | 0.133849 | 4.00E-04 | 9.22E-13 | 0.22 | 11.579633 |
| rs1136201 | A | G | 0.0046 | -0.0016887 | 0.7521 | 0.751859 | 3.00E-04 | 6.47E-46 | 0.27 | 87.678341 |
| rs1137844 | C | G | -0.0021 | 0.0023419 | 0.6841 | 0.686965 | 3.00E-04 | 7.86E-12 | 0.1 | 21.178909 |
| rs11564722 | T | C | 0.0041 | -0.0015287 | 0.2406 | 0.238771 | 3.00E-04 | 4.11E-35 | 0.33 | 68.257452 |
| rs11616030 | A | C | -0.004 | -0.0024419 | 0.9119 | 0.913683 | 5.00E-04 | 2.31E-16 | 0.3 | 10.283399 |
| rs11642550 | A | G | 0.0018 | 0.0012413 | 0.303 | 0.303871 | 3.00E-04 | 2.82E-09 | 0.39 | 15.205952 |
| rs11694902 | A | G | 0.0041 | -3.02E-03 | 0.1397 | 0.139906 | 4.00E-04 | 1.46E-25 | 0.11 | 25.254229 |
| rs11702255 | A | G | -0.0017 | 0.0002451 | 0.3692 | 0.376039 | 3.00E-04 | 9.66E-09 | 0.86 | 14.956994 |
| rs11750211 | T | C | -0.002 | -0.0013945 | 0.2723 | 0.274096 | 3.00E-04 | 1.01E-10 | 0.35 | 17.613848 |
| rs11759908 | T | C | -0.0027 | 0.0012851 | 0.4519 | 0.455767 | 3.00E-04 | 2.57E-21 | 0.34 | 40.126719 |
| rs11856921 | A | C | 0.0027 | 0.0003782 | 0.4297 | 0.428785 | 3.00E-04 | 5.00E-23 | 0.78 | 39.700872 |
| rs11864372 | T | C | 0.0025 | -0.0005014 | 0.5018 | 0.505538 | 3.00E-04 | 1.88E-20 | 0.7 | 34.722904 |
| rs11894252 | T | C | -0.0015 | -0.0015837 | 0.412 | 0.406159 | 3.00E-04 | 1.76E-08 | 0.24 | 12.112922 |
| rs11894444 | A | G | -0.0015 | 0.0016489 | 0.5293 | 0.530932 | 3.00E-04 | 4.43E-08 | 0.22 | 12.457205 |
| rs1214761 | A | G | 0.0024 | -0.0080084 | 0.3259 | 0.319707 | 3.00E-04 | 3.53E-17 | 1.5E-08 | 28.120948 |
| rs12163971 | A | C | -0.0027 | 0.0032844 | 0.1625 | 0.166422 | 4.00E-04 | 2.56E-13 | 0.063 | 12.401671 |
| rs12432201 | T | C | 0.0022 | 0.0014724 | 0.6926 | 0.695365 | 3.00E-04 | 1.98E-13 | 0.31 | 22.899618 |
| rs12449763 | A | T | -0.0022 | -0.001138 | 0.7822 | 0.776634 | 3.00E-04 | 1.20E-10 | 0.48 | 18.323802 |
| rs12475244 | T | C | 0.0022 | -0.0001221 | 0.6325 | 0.634735 | 3.00E-04 | 4.01E-14 | 0.93 | 25.001189 |
| rs12476256 | T | C | -0.0026 | -9.104E-06 | 0.4185 | 0.417439 | 3.00E-04 | 8.33E-21 | 0.99 | 36.559 |
| rs12520984 | C | G | 0.0023 | 0.0005181 | 0.3214 | 0.311686 | 3.00E-04 | 6.48E-16 | 0.72 | 25.63971 |
| rs12781024 | A | T | 0.0017 | 2.40E-03 | 0.5078 | 0.50152 | 3.00E-04 | 4.87E-10 | 0.07 | 16.051873 |
| rs12950549 | A | G | 0.0017 | 0.0015255 | 0.6459 | 0.65127 | 3.00E-04 | 2.81E-09 | 0.27 | 14.688655 |
| rs13002079 | T | C | -0.003 | 0.0008119 | 0.6531 | 0.656718 | 3.00E-04 | 2.87E-26 | 0.56 | 45.314033 |
| rs13032786 | C | G | 0.0032 | 0.0003724 | 0.6961 | 0.702082 | 3.00E-04 | 3.24E-27 | 0.8 | 48.140404 |
| rs13047277 | T | C | 0.0024 | 0.0008394 | 0.71 | 0.7 | 3.00E-04 | 7.42E-16 | 0.56 | 26.355839 |
| rs13157326 | A | G | -0.0024 | -0.0038449 | 0.4722 | 0.466075 | 3.00E-04 | 1.60E-16 | 0.004 | 31.902027 |
| rs1321917 | C | G | -0.0024 | -0.0004754 | 0.4043 | 0.408698 | 3.00E-04 | 3.96E-18 | 0.72 | 30.828598 |
| rs13227214 | C | G | -0.0027 | -0.0003711 | 0.4561 | 0.453225 | 3.00E-04 | 1.71E-23 | 0.78 | 40.189321 |
| rs13230509 | C | G | -0.0064 | -0.0003729 | 0.6906 | 0.691899 | 3.00E-04 | 1.03E-88 | 0.8 | 194.52597 |
| rs13247734 | A | G | 0.0018 | -0.0007452 | 0.5647 | 0.568901 | 3.00E-04 | 2.34E-11 | 0.58 | 17.698878 |
| rs1438898 | A | C | 0.0017 | 0.0027064 | 0.7499 | 0.748679 | 3.00E-04 | 2.32E-08 | 0.076 | 12.044998 |
| rs1458038 | T | C | 0.0031 | -0.0021227 | 0.2957 | 0.293214 | 3.00E-04 | 6.88E-26 | 0.14 | 44.477284 |
| rs151245 | T | G | -0.002 | 0.0002452 | 0.6016 | 0.600734 | 3.00E-04 | 4.80E-13 | 0.86 | 21.305071 |
| rs1519102 | C | G | 0.0018 | -0.0015802 | 0.6884 | 0.687772 | 3.00E-04 | 1.74E-09 | 0.27 | 15.444598 |
| rs1541938 | T | C | 0.0029 | 0.0013876 | 0.2637 | 0.256908 | 3.00E-04 | 1.25E-20 | 0.36 | 36.288018 |
| rs1542337 | A | G | -0.0021 | -0.002278 | 0.4001 | 0.406625 | 3.00E-04 | 9.54E-14 | 0.091 | 23.522463 |
| rs1548945 | T | C | 0.0038 | 0.0009078 | 0.4145 | 0.42024 | 3.00E-04 | 1.96E-41 | 0.5 | 77.88233 |
| rs16823029 | A | C | -0.0053 | 0.0010588 | 0.0814 | 0.080522 | 5.00E-04 | 3.35E-26 | 0.66 | 16.80347 |
| rs16843860 | A | G | 0.0019 | -0.0010823 | 0.7175 | 0.713094 | 3.00E-04 | 3.39E-10 | 0.46 | 16.260774 |
| rs169046 | T | C | 0.0021 | 0.0044078 | 0.5645 | 0.568 | 3.00E-04 | 7.03E-15 | 0.00094 | 24.092826 |
| rs17050272 | A | G | -0.0029 | 0.0008955 | 0.4193 | 0.411021 | 3.00E-04 | 2.01E-25 | 0.5 | 45.507082 |
| rs17256228 | T | C | -0.0056 | 0.001938 | 0.1084 | 0.107916 | 4.00E-04 | 9.45E-39 | 0.36 | 37.887935 |
| rs17413465 | A | C | 0.0025 | -0.0006656 | 0.1849 | 0.189292 | 3.00E-04 | 1.01E-12 | 0.69 | 20.932616 |
| rs17563 | A | G | 0.0018 | 0.0011118 | 0.4292 | 0.425065 | 3.00E-04 | 5.61E-11 | 0.4 | 17.639365 |
| rs1757915 | A | G | 0.0024 | 8.31E-04 | 0.3532 | 0.355959 | 3.00E-04 | 7.84E-17 | 0.55 | 29.242363 |
| rs1772976 | T | C | 0.0017 | 0.0005764 | 0.5183 | 0.515431 | 3.00E-04 | 1.14E-09 | 0.66 | 16.034272 |
| rs1801251 | A | G | 0.0019 | -0.0029371 | 0.3571 | 0.363776 | 3.00E-04 | 7.64E-12 | 0.032 | 18.417686 |
| rs1883991 | A | C | -0.0028 | 0.0020935 | 0.6805 | 0.671735 | 3.00E-04 | 2.48E-22 | 0.14 | 37.880706 |
| rs1887251 | T | C | 0.0029 | -0.0003347 | 0.3626 | 0.365227 | 3.00E-04 | 1.69E-25 | 0.81 | 43.195764 |
| rs2053196 | T | C | 0.0018 | -0.0007892 | 0.7876 | 0.794572 | 3.00E-04 | 3.63E-08 | 0.63 | 12.04473 |
| rs2068888 | A | G | -0.0031 | -0.0045294 | 0.4504 | 0.447761 | 3.00E-04 | 7.61E-30 | 0.00063 | 52.866186 |
| rs2076668 | A | G | -0.0039 | 4.78E-05 | 0.3847 | 0.380111 | 3.00E-04 | 6.91E-43 | 0.97 | 80.012814 |
| rs219781 | T | G | 0.0024 | -0.0013867 | 0.2567 | 0.263666 | 3.00E-04 | 3.28E-15 | 0.35 | 24.4236 |
| rs223308 | A | G | -0.003 | 0.0019164 | 0.5179 | 0.515566 | 3.00E-04 | 4.24E-28 | 0.15 | 49.938302 |
| rs2235826 | A | T | -0.0033 | 0.0003244 | 0.812 | 0.814486 | 4.00E-04 | 2.05E-20 | 0.85 | 20.780687 |
| rs2267372 | A | G | 0.0024 | -0.0005483 | 0.4001 | 0.399513 | 3.00E-04 | 9.33E-18 | 0.68 | 30.723438 |
| rs2289746 | T | C | -0.0017 | -0.0009685 | 0.3361 | 0.332491 | 3.00E-04 | 4.15E-09 | 0.49 | 14.330517 |
| rs2302316 | A | G | 0.0018 | -0.001493 | 0.2353 | 0.229622 | 3.00E-04 | 3.30E-08 | 0.35 | 12.955383 |
| rs2306593 | T | C | 0.0017 | -0.0008646 | 0.489 | 0.487966 | 3.00E-04 | 1.13E-09 | 0.51 | 16.048009 |
| rs2625166 | A | G | 0.0016 | -0.0010911 | 0.4308 | 0.432057 | 3.00E-04 | 4.61E-08 | 0.42 | 13.949968 |
| rs267738 | T | G | -0.0053 | 0.0003365 | 0.7849 | 0.779096 | 3.00E-04 | 2.38E-57 | 0.83 | 105.39953 |
| rs2694158 | T | G | 0.0019 | 0.001739 | 0.3801 | 0.380658 | 3.00E-04 | 7.96E-12 | 0.2 | 18.902598 |
| rs2783971 | A | C | -0.0027 | -0.003796 | 0.4729 | 0.466042 | 3.00E-04 | 5.90E-24 | 0.0041 | 40.382569 |
| rs278941 | A | G | -0.0019 | -0.0015356 | 0.7179 | 0.728755 | 3.00E-04 | 3.74E-10 | 0.3 | 16.246802 |
| rs2815367 | A | G | -0.0019 | -0.0012732 | 0.6502 | 0.648286 | 3.00E-04 | 5.21E-11 | 0.36 | 18.246034 |
| rs2823139 | A | G | -0.0032 | -0.0002733 | 0.3387 | 0.33838 | 3.00E-04 | 3.03E-29 | 0.85 | 50.970905 |
| rs2834317 | A | G | -0.0037 | 0.0016579 | 0.1521 | 0.153228 | 4.00E-04 | 2.80E-22 | 0.37 | 22.069678 |
| rs28493806 | A | G | -0.0015 | -0.0009343 | 0.6243 | 0.630038 | 3.00E-04 | 4.50E-08 | 0.49 | 11.727589 |
| rs2875930 | T | C | -0.0019 | 0.0007508 | 0.2444 | 0.249424 | 3.00E-04 | 1.19E-09 | 0.62 | 14.814718 |
| rs2945914 | A | C | -0.0018 | 0.0019015 | 0.3608 | 0.359448 | 3.00E-04 | 2.25E-10 | 0.17 | 16.605123 |
| rs2960455 | A | G | 0.0043 | 0.000473 | 0.7148 | 0.707734 | 3.00E-04 | 2.05E-46 | 0.74 | 83.771025 |
| rs303938 | T | G | -0.0027 | -0.0022521 | 0.5975 | 0.606158 | 3.00E-04 | 8.65E-22 | 0.096 | 38.961422 |
| rs3118918 | A | G | -0.0027 | -0.0033018 | 0.7897 | 0.788565 | 3.00E-04 | 5.27E-16 | 0.041 | 26.904641 |
| rs3125056 | T | C | 0.0066 | 0.0016763 | 0.8716 | 0.861088 | 4.00E-04 | 7.52E-60 | 0.38 | 60.940445 |
| rs34049619 | A | G | -0.0024 | 0.0008786 | 0.1685 | 0.157574 | 4.00E-04 | 4.58E-11 | 0.63 | 10.087839 |
| rs34084915 | A | G | -0.0024 | -0.0018343 | 0.8249 | 0.830114 | 4.00E-04 | 3.69E-11 | 0.3 | 10.399766 |
| rs34188292 | C | G | -0.0019 | -0.0030741 | 0.2651 | 0.271303 | 3.00E-04 | 9.37E-09 | 0.039 | 15.629265 |
| rs34642860 | T | C | 0.0021 | -0.0036987 | 0.3054 | 0.312538 | 3.00E-04 | 1.44E-12 | 0.0094 | 20.789211 |
| rs34753685 | A | G | -0.0038 | 0.0009979 | 0.4443 | 0.447178 | 3.00E-04 | 9.59E-44 | 0.45 | 79.232762 |
| rs35072105 | A | G | -0.002 | -0.0039434 | 0.5391 | 0.527635 | 3.00E-04 | 7.74E-13 | 0.0028 | 22.08677 |
| rs35320690 | T | C | -0.0026 | 0.0001148 | 0.7225 | 0.724944 | 3.00E-04 | 1.79E-17 | 0.94 | 30.11946 |
| rs35629566 | C | G | 0.0028 | 0.0045841 | 0.8244 | 0.820272 | 4.00E-04 | 5.40E-14 | 0.0081 | 14.187107 |
| rs35917667 | A | C | 0.0022 | 0.0034674 | 0.2395 | 0.240776 | 3.00E-04 | 8.98E-12 | 0.025 | 19.590485 |
| rs35969577 | T | G | -0.0061 | -0.0014413 | 0.4162 | 0.411033 | 3.00E-04 | 4.43E-105 | 0.28 | 200.95526 |
| rs36034566 | A | C | -0.0016 | 4.12E-04 | 0.5235 | 0.516122 | 3.00E-04 | 4.11E-09 | 0.75 | 14.190978 |
| rs3774292 | A | T | 0.0029 | -0.0012233 | 0.6827 | 0.686343 | 3.00E-04 | 5.37E-23 | 0.39 | 40.485556 |
| rs3791221 | A | G | 0.0022 | 0.0016777 | 0.6508 | 0.649399 | 3.00E-04 | 1.39E-14 | 0.22 | 24.443553 |
| rs3795503 | T | C | 0.0024 | -0.0001441 | 0.3218 | 0.315538 | 3.00E-04 | 1.57E-16 | 0.92 | 27.936051 |
| rs3797537 | A | G | 0.0021 | -0.0005025 | 0.7134 | 0.717102 | 3.00E-04 | 1.88E-12 | 0.73 | 20.037483 |
| rs3812036 | T | C | -0.0071 | -0.0025183 | 0.252 | 0.244368 | 3.00E-04 | 3.89E-109 | 0.1 | 211.2014 |
| rs3814828 | A | G | 0.0021 | 0.0007469 | 0.3841 | 0.386863 | 3.00E-04 | 4.69E-13 | 0.59 | 23.184074 |
| rs3824081 | T | C | -0.0015 | 0.0033207 | 0.4783 | 0.474739 | 3.00E-04 | 1.91E-08 | 0.012 | 12.476586 |
| rs3850625 | A | G | 0.005 | 0.0067254 | 0.119 | 0.118718 | 4.00E-04 | 1.52E-31 | 0.00095 | 32.763191 |
| rs3904600 | C | G | 0.003 | -0.0036396 | 0.3706 | 0.369528 | 3.00E-04 | 3.96E-26 | 0.0078 | 46.653203 |
| rs3925584 | T | C | -0.0053 | 0.0010662 | 0.5476 | 0.544461 | 3.00E-04 | 9.31E-86 | 0.42 | 154.66473 |
| rs429358 | T | C | -0.0031 | -0.0017298 | 0.8429 | 0.84458 | 4.00E-04 | 5.13E-16 | 0.34 | 15.907124 |
| rs4434960 | A | T | 0.0022 | -0.0026064 | 0.6153 | 0.612674 | 3.00E-04 | 2.46E-15 | 0.055 | 25.459631 |
| rs4493120 | C | G | -0.0032 | 0.0007159 | 0.5409 | 0.541482 | 3.00E-04 | 1.71E-30 | 0.59 | 56.5113 |
| rs4567937 | A | G | -0.0034 | 0.0032921 | 0.3182 | 0.31625 | 3.00E-04 | 8.91E-31 | 0.02 | 55.734709 |
| rs4617830 | A | C | 0.0024 | -0.0001715 | 0.7816 | 0.784627 | 3.00E-04 | 2.58E-13 | 0.91 | 21.850216 |
| rs4705067 | C | G | -0.0019 | -0.0013049 | 0.2087 | 0.205807 | 3.00E-04 | 1.09E-08 | 0.42 | 13.248392 |
| rs4735334 | A | G | -0.0017 | 0.0009009 | 0.6982 | 0.695233 | 3.00E-04 | 6.28E-09 | 0.53 | 13.532854 |
| rs4744712 | A | C | -0.0049 | -0.0023339 | 0.399 | 0.39889 | 3.00E-04 | 1.26E-67 | 0.083 | 127.96214 |
| rs4859682 | A | C | -0.0079 | -0.0031485 | 0.4477 | 0.458123 | 3.00E-04 | 4.10E-187 | 0.017 | 343.04516 |
| rs4871905 | C | G | -0.0049 | 0.0034553 | 0.4165 | 0.413403 | 3.00E-04 | 7.41E-72 | 0.0098 | 129.6853 |
| rs4946932 | A | C | 0.0028 | 0.0014498 | 0.3022 | 0.292717 | 3.00E-04 | 4.22E-21 | 0.32 | 36.74041 |
| rs511893 | T | G | 0.0017 | -0.0025858 | 0.5794 | 0.579957 | 3.00E-04 | 4.83E-09 | 0.059 | 15.650888 |
| rs55658481 | A | G | 0.002 | 0.0005234 | 0.3399 | 0.340566 | 3.00E-04 | 8.04E-13 | 0.71 | 19.944178 |
| rs55924910 | C | G | 0.0026 | -0.0023182 | 0.8452 | 0.838816 | 4.00E-04 | 2.92E-12 | 0.2 | 11.055823 |
| rs56065557 | C | G | -0.0032 | 0.0006587 | 0.3109 | 0.311379 | 3.00E-04 | 2.92E-27 | 0.65 | 48.754043 |
| rs56252444 | T | G | -0.0031 | -0.0005124 | 0.6208 | 0.610144 | 3.00E-04 | 9.08E-29 | 0.7 | 50.274967 |
| rs59646751 | T | G | -0.0023 | 0.0016072 | 0.3102 | 0.309941 | 3.00E-04 | 1.02E-14 | 0.26 | 25.154643 |
| rs60503594 | T | C | -0.0022 | -0.000525 | 0.6616 | 0.664087 | 3.00E-04 | 6.21E-15 | 0.71 | 24.080652 |
| rs6055748 | A | G | -0.0018 | 0.000141 | 0.6914 | 0.692079 | 3.00E-04 | 6.31E-10 | 0.92 | 15.362559 |
| rs611760 | A | C | -0.0019 | -0.0007128 | 0.812 | 0.816273 | 3.00E-04 | 3.08E-08 | 0.68 | 12.246529 |
| rs6127099 | A | T | -0.0048 | 0.0008579 | 0.7204 | 0.721476 | 3.00E-04 | 3.83E-52 | 0.57 | 103.13939 |
| rs61988865 | A | C | 0.0021 | 0.0015193 | 0.2228 | 0.217588 | 3.00E-04 | 6.04E-10 | 0.34 | 16.969949 |
| rs6458868 | T | C | -0.002 | -0.0005147 | 0.6471 | 0.645965 | 3.00E-04 | 5.18E-13 | 0.71 | 20.299178 |
| rs6467958 | T | C | 0.0024 | -0.0024872 | 0.273 | 0.267152 | 3.00E-04 | 2.19E-15 | 0.095 | 25.40488 |
| rs6501179 | T | C | 0.0019 | -0.0005554 | 0.5601 | 0.560077 | 3.00E-04 | 3.60E-12 | 0.68 | 19.766142 |
| rs6546861 | T | C | 0.0063 | -0.0007745 | 0.229 | 0.228767 | 3.00E-04 | 3.74E-85 | 0.62 | 155.74888 |
| rs6679160 | A | G | 0.0029 | -0.0015094 | 0.1284 | 0.125692 | 4.00E-04 | 4.97E-12 | 0.45 | 11.765015 |
| rs6708702 | A | G | -0.0019 | 0.0004645 | 0.3019 | 0.282303 | 3.00E-04 | 4.61E-10 | 0.75 | 16.907597 |
| rs6784541 | A | G | -0.0021 | 0.0011551 | 0.5361 | 0.5312 | 3.00E-04 | 6.06E-15 | 0.38 | 24.372829 |
| rs6899133 | T | C | 0.0016 | -0.0021045 | 0.5361 | 0.541168 | 3.00E-04 | 9.10E-09 | 0.11 | 14.148255 |
| rs693906 | C | G | -0.004 | -0.0074671 | 0.1491 | 0.157941 | 4.00E-04 | 2.02E-25 | 0.000041 | 25.374429 |
| rs6948759 | T | C | -0.0024 | 0.0017786 | 0.2038 | 0.195406 | 3.00E-04 | 6.31E-13 | 0.28 | 20.77038 |
| rs6968865 | A | T | -0.0026 | -0.0006135 | 0.3711 | 0.368618 | 3.00E-04 | 2.87E-20 | 0.65 | 35.060736 |
| rs700753 | C | G | 0.0034 | 0.0005344 | 0.3435 | 0.346592 | 3.00E-04 | 2.30E-32 | 0.7 | 57.933663 |
| rs7019647 | A | G | 0.0021 | 0.0026086 | 0.7347 | 0.730256 | 3.00E-04 | 1.85E-12 | 0.079 | 19.102085 |
| rs7036795 | T | C | -0.0022 | -3.234E-05 | 0.8008 | 0.80277 | 3.00E-04 | 2.22E-10 | 0.98 | 17.157452 |
| rs7096715 | T | C | -0.0019 | 0.0020496 | 0.4217 | 0.423396 | 3.00E-04 | 8.99E-12 | 0.13 | 19.564064 |
| rs7114014 | T | C | -0.0018 | -0.0002592 | 0.6491 | 0.648798 | 3.00E-04 | 2.86E-10 | 0.85 | 16.399617 |
| rs71512638 | A | T | -0.0019 | -0.0013796 | 0.7996 | 0.800279 | 3.00E-04 | 2.65E-08 | 0.41 | 12.854935 |
| rs717614 | C | G | -0.002 | 0.003709 | 0.5214 | 0.52268 | 3.00E-04 | 3.07E-13 | 0.0052 | 22.181961 |
| rs7217845 | A | G | 0.0018 | -0.0001748 | 0.3318 | 0.32443 | 3.00E-04 | 4.78E-10 | 0.9 | 15.963253 |
| rs7247977 | T | C | -0.0049 | -0.0004485 | 0.6056 | 0.603707 | 3.00E-04 | 2.40E-71 | 0.74 | 127.45494 |
| rs72695707 | T | C | 0.002 | -0.0007978 | 0.2177 | 0.216208 | 3.00E-04 | 2.00E-09 | 0.62 | 15.138572 |
| rs72912510 | A | G | -0.0026 | -0.0051048 | 0.204 | 0.197097 | 3.00E-04 | 1.16E-13 | 0.0023 | 24.394229 |
| rs7298123 | T | C | 0.0019 | -0.003846 | 0.8002 | 0.801489 | 3.00E-04 | 1.24E-08 | 0.02 | 12.826064 |
| rs7307046 | A | G | -0.0016 | -0.0022319 | 0.4131 | 0.416148 | 3.00E-04 | 1.21E-08 | 0.095 | 13.792782 |
| rs73116888 | T | C | -0.004 | 0.0016908 | 0.0995 | 0.089854 | 5.00E-04 | 1.02E-17 | 0.46 | 11.468876 |
| rs73119035 | A | G | 0.0034 | -0.0003187 | 0.8668 | 0.864365 | 4.00E-04 | 4.04E-17 | 0.87 | 16.68389 |
| rs73119306 | A | G | -0.0031 | -0.0003683 | 0.7547 | 0.755482 | 3.00E-04 | 7.04E-23 | 0.81 | 39.536572 |
| rs74183052 | T | C | -0.0019 | 0.0034134 | 0.3572 | 0.356042 | 3.00E-04 | 2.54E-10 | 0.014 | 18.419978 |
| rs7492724 | A | G | 0.002 | -0.000453 | 0.7082 | 0.70708 | 3.00E-04 | 7.29E-12 | 0.76 | 18.369434 |
| rs75094798 | A | T | 0.004 | 0.0021064 | 0.0935 | 0.09463 | 5.00E-04 | 7.21E-16 | 0.35 | 10.849088 |
| rs7514450 | T | C | 0.0022 | -0.0028811 | 0.4277 | 0.427085 | 3.00E-04 | 1.03E-15 | 0.031 | 26.327303 |
| rs7514579 | A | C | -0.0026 | -0.0010492 | 0.7688 | 0.766992 | 3.00E-04 | 5.25E-15 | 0.5 | 26.70214 |
| rs7516435 | A | G | 0.0031 | -0.001669 | 0.6884 | 0.685458 | 3.00E-04 | 5.06E-26 | 0.24 | 45.810827 |
| rs7528419 | A | G | 0.0033 | -0.0053975 | 0.7787 | 0.776815 | 3.00E-04 | 2.48E-24 | 0.00065 | 41.704616 |
| rs753381 | T | C | -0.0016 | 0.0051703 | 0.4542 | 0.448247 | 3.00E-04 | 2.79E-09 | 0.000098 | 14.10306 |
| rs7536433 | T | C | 0.0025 | 0.0002098 | 0.2111 | 0.213052 | 3.00E-04 | 1.88E-13 | 0.9 | 23.130597 |
| rs754469 | A | G | -0.0024 | 0.0010247 | 0.5205 | 0.520626 | 3.00E-04 | 2.25E-17 | 0.47 | 31.947161 |
| rs7565830 | A | G | -0.0018 | -0.0002639 | 0.7246 | 0.729846 | 3.00E-04 | 2.81E-09 | 0.86 | 14.368125 |
| rs760418 | C | G | -0.002 | 0.0001987 | 0.5249 | 0.519339 | 3.00E-04 | 7.95E-14 | 0.88 | 22.167555 |
| rs76273615 | A | G | 0.003 | 0.0010947 | 0.8691 | 0.877018 | 4.00E-04 | 1.81E-13 | 0.59 | 12.798722 |
| rs7740107 | A | T | 0.0029 | 0.0014522 | 0.738 | 0.734861 | 3.00E-04 | 7.19E-22 | 0.33 | 36.137317 |
| rs7760528 | T | C | 0.0019 | 0.0009341 | 0.68 | 0.680996 | 3.00E-04 | 2.40E-10 | 0.51 | 17.456624 |
| rs77924615 | A | G | 0.0106 | 0.0008122 | 0.1984 | 0.197991 | 3.00E-04 | 2.15E-200 | 0.63 | 397.25514 |
| rs79346194 | A | G | -0.0021 | 0.0016998 | 0.6921 | 0.694683 | 3.00E-04 | 7.07E-13 | 0.24 | 20.883957 |
| rs7953798 | T | G | -0.0045 | 0.0020623 | 0.09 | 0.087614 | 5.00E-04 | 6.43E-21 | 0.38 | 13.267949 |
| rs79760705 | T | G | 0.0062 | 0.0009642 | 0.1115 | 0.113314 | 4.00E-04 | 1.08E-46 | 0.64 | 47.604216 |
| rs80282103 | A | T | 0.0083 | 0.0015132 | 0.9181 | 0.916175 | 5.00E-04 | 2.61E-62 | 0.53 | 41.441658 |
| rs8062982 | C | G | -0.0022 | -0.0040565 | 0.424 | 0.429497 | 3.00E-04 | 6.06E-15 | 0.0028 | 26.268283 |
| rs807624 | T | G | 0.0036 | -0.0019233 | 0.3489 | 0.358391 | 3.00E-04 | 3.09E-37 | 0.16 | 65.428745 |
| rs8096658 | C | G | 0.0052 | -0.0014959 | 0.5114 | 0.512319 | 3.00E-04 | 6.40E-71 | 0.26 | 150.16629 |
| rs81205 | A | C | 0.003 | 0.0019497 | 0.5375 | 0.537805 | 3.00E-04 | 3.32E-26 | 0.15 | 49.721113 |
| rs849086 | A | G | 0.0019 | -0.0013983 | 0.2802 | 0.2805 | 3.00E-04 | 2.04E-10 | 0.34 | 16.180085 |
| rs871664 | T | C | 0.0015 | 0.0002455 | 0.4808 | 0.473402 | 3.00E-04 | 2.27E-08 | 0.85 | 12.481698 |
| rs8866 | C | G | -0.0017 | -0.0008841 | 0.6044 | 0.595618 | 3.00E-04 | 1.93E-09 | 0.51 | 15.355779 |
| rs9333592 | T | C | 0.0053 | 0.0048673 | 0.0779 | 0.077309 | 5.00E-04 | 9.51E-22 | 0.052 | 16.142222 |
| rs9397738 | A | G | 0.0029 | 0.0023699 | 0.8588 | 0.85732 | 4.00E-04 | 1.31E-13 | 0.21 | 12.747863 |
| rs941352 | A | C | 0.0027 | 0.0013997 | 0.796 | 0.802211 | 4.00E-04 | 4.94E-14 | 0.4 | 14.797431 |
| rs952151 | A | G | -0.0023 | -0.0015243 | 0.2341 | 0.237855 | 3.00E-04 | 3.86E-13 | 0.32 | 21.077781 |
| rs9521720 | C | G | -0.0021 | 0.0017023 | 0.4005 | 0.398512 | 3.00E-04 | 1.17E-13 | 0.21 | 23.53028 |
| rs967532 | A | G | -0.0019 | -0.0024187 | 0.6281 | 0.62674 | 3.00E-04 | 1.50E-11 | 0.076 | 18.739453 |
| rs9823161 | A | G | 0.0025 | 0.0009008 | 0.6871 | 0.69425 | 3.00E-04 | 4.35E-15 | 0.53 | 29.861049 |
| rs983310 | C | G | -0.0017 | -0.0018248 | 0.2465 | 0.244065 | 3.00E-04 | 4.25E-08 | 0.24 | 11.928609 |
| rs9868185 | A | G | 0.0029 | 0.0029963 | 0.5316 | 0.519651 | 3.00E-04 | 3.86E-26 | 0.023 | 46.537667 |
| rs9894634 | T | C | -0.0019 | 0.0010245 | 0.6003 | 0.598145 | 3.00E-04 | 2.84E-12 | 0.45 | 19.248843 |
| rs9905761 | T | C | 0.006 | 0.0011128 | 0.1878 | 0.184907 | 3.00E-04 | 5.77E-67 | 0.51 | 122.03952 |
| rs9925050 | C | G | -0.0019 | 0.0015847 | 0.7596 | 0.769232 | 3.00E-04 | 1.82E-09 | 0.31 | 14.649391 |
| rs9932625 | A | G | -0.0033 | -0.0005174 | 0.2295 | 0.229505 | 3.00E-04 | 4.66E-25 | 0.74 | 42.794538 |

8 SNPs (rs10224002, rs223308, rs35320690, rs429358, rs4434960, rs4946932, rs77924615, rs9932625) that were removed from PhenoScanner step, 7 SNPs (rs12781024, rs13227214, rs4493120, rs717614, rs760418, rs8062982, rs8096658) that were removed from harmonization step, and 3 SNPs (rs1214761, rs693906, rs753381) that were removed from MR-PRESSO step.

**Table S5** Effect estimates for the associations of the genetic variants with eGFR_cys and hearing loss.

| **SNP** | **effect_allele** | **other_allele** | **beta.exposure** | **beta.outcome** | **eaf.exposure** | **eaf.outcome** | **se.exposure** | **pval.exposure** | **pval.outcome** | **F_statisic** |
| --- | --- | --- | --- | --- | --- | --- | --- | --- | --- | --- |
| rs10010367 | A | C | 0.0037 | -0.000105 | 0.6029 | 0.601854 | 5.00E-04 | 5.93E-15 | 0.94 | 26.22 |
| rs10051765 | T | C | 0.0077 | 0.0006837 | 0.6664 | 0.668398 | 5.00E-04 | 6.21E-56 | 0.63 | 105.47 |
| rs10092747 | T | C | 0.0035 | -0.000949 | 0.8096 | 0.810718 | 6.00E-04 | 3.19E-09 | 0.57 | 10.49 |
| rs1017545 | T | C | -0.004 | -0.0022 | 0.3577 | 0.353152 | 5.00E-04 | 1.471E-16 | 0.11 | 29.41 |
| rs10189685 | A | G | -0.0065 | 0.0017969 | 0.293 | 0.29601 | 5.00E-04 | 4.76E-38 | 0.21 | 70.03 |
| rs10206899 | T | C | -0.0051 | 0.0011299 | 0.7777 | 0.778068 | 6.00E-04 | 2.62E-20 | 0.48 | 24.98 |
| rs10224210 | T | C | 0.0092 | 0.0034335 | 0.7224 | 0.718022 | 5.00E-04 | 2.04E-72 | 0.019 | 135.83 |
| rs10782784 | C | G | -0.0029 | 0.0010949 | 0.5756 | 0.579403 | 5.00E-04 | 6.76E-10 | 0.41 | 16.44 |
| rs10806987 | T | C | -0.0027 | -0.000173 | 0.4029 | 0.394019 | 5.00E-04 | 8.12E-09 | 0.9 | 14.03 |
| rs10821907 | T | C | 0.0042 | 0.002538 | 0.1797 | 0.17862 | 6.00E-04 | 2.93E-12 | 0.15 | 14.45 |
| rs10824218 | A | T | -0.0034 | -0.006939 | 0.5716 | 0.567085 | 5.00E-04 | 1.72E-13 | 2.3E-07 | 22.65 |
| rs10838681 | A | G | 0.0033 | -0.00087 | 0.2737 | 0.268173 | 5.00E-04 | 3.07E-10 | 0.56 | 17.32 |
| rs10846156 | T | G | -0.0056 | 0.0029742 | 0.7977 | 0.798035 | 6.00E-04 | 1.48E-22 | 0.071 | 28.12 |
| rs10857147 | A | T | -0.0029 | 0.0021321 | 0.7112 | 0.70807 | 5.00E-04 | 9.44E-09 | 0.14 | 13.82 |
| rs10896037 | A | G | 0.0055 | 0.0004389 | 0.6593 | 0.655976 | 5.00E-04 | 1.06E-29 | 0.75 | 54.37 |
| rs10901812 | A | G | 0.0062 | -0.001902 | 0.263 | 0.265564 | 5.00E-04 | 1.31E-32 | 0.2 | 59.61 |
| rs10933713 | C | G | 0.0038 | 0.0014071 | 0.6902 | 0.702788 | 5.00E-04 | 5.81E-14 | 0.33 | 24.70 |
| rs10993074 | T | C | -0.0028 | 2.08E-04 | 0.3163 | 0.32025 | 5.00E-04 | 2.29E-08 | 0.88 | 13.56 |
| rs11072567 | A | G | 0.0059 | 0.0008502 | 0.4906 | 0.487799 | 5.00E-04 | 2.48E-38 | 0.52 | 69.61 |
| rs11115974 | A | G | -0.003 | -0.000462 | 0.5491 | 0.549667 | 5.00E-04 | 1.42E-10 | 0.73 | 17.83 |
| rs1122750 | A | G | -0.0032 | 0.0017721 | 0.2782 | 0.274985 | 5.00E-04 | 6.73E-10 | 0.23 | 16.45 |
| rs1131285 | C | G | -0.0058 | -0.004431 | 0.3521 | 0.35683 | 5.00E-04 | 5.30E-33 | 0.0013 | 61.40 |
| rs113367286 | T | C | 0.0028 | 0.0004607 | 0.2752 | 0.279855 | 5.00E-04 | 3.47E-08 | 0.75 | 12.51 |
| rs11629006 | T | C | 0.0039 | 0.0003555 | 0.8143 | 0.81833 | 6.00E-04 | 4.26E-11 | 0.84 | 12.78 |
| rs11651885 | A | G | -0.005 | -0.001987 | 0.7713 | 0.78158 | 6.00E-04 | 2.16E-19 | 0.21 | 24.50 |
| rs11768336 | T | C | -0.0041 | 0.0005796 | 0.2595 | 0.249281 | 5.00E-04 | 4.45E-15 | 0.7 | 25.84 |
| rs11856921 | A | C | 0.0035 | 0.0003782 | 0.4251 | 0.428785 | 5.00E-04 | 3.92E-14 | 0.78 | 23.95 |
| rs11860535 | T | C | 0.0047 | 0.0032224 | 0.7106 | 0.709593 | 5.00E-04 | 1.64E-19 | 0.031 | 36.34 |
| rs12045944 | A | C | 0.0026 | -0.000111 | 0.5405 | 0.542192 | 5.00E-04 | 2.07E-08 | 0.93 | 13.43 |
| rs12136896 | A | G | 0.0028 | 0.0008787 | 0.3548 | 0.356842 | 5.00E-04 | 1.09E-08 | 0.52 | 14.36 |
| rs12536253 | C | G | 0.0042 | -0.002562 | 0.2478 | 0.248278 | 5.00E-04 | 1.84E-15 | 0.094 | 26.31 |
| rs12601991 | T | G | -0.003 | 0.0003664 | 0.4048 | 0.406352 | 5.00E-04 | 1.01E-10 | 0.79 | 17.35 |
| rs1260326 | T | C | 0.004 | 0.0020343 | 0.3949 | 0.392533 | 0.0005 | 2.81E-17 | 0.13 | 30.59 |
| rs12713261 | T | C | 0.0031 | -0.001996 | 0.6476 | 0.648673 | 5.00E-04 | 9.84E-11 | 0.15 | 17.55 |
| rs12751255 | A | C | -0.0029 | 0.0003298 | 0.6296 | 0.634461 | 5.00E-04 | 9.47E-10 | 0.81 | 15.69 |
| rs12799529 | T | C | -0.0031 | 0.002587 | 0.7598 | 0.756138 | 5.00E-04 | 7.49E-09 | 0.093 | 14.03 |
| rs13000374 | A | G | -0.003 | -0.000215 | 0.2736 | 0.270278 | 5.00E-04 | 4.75E-09 | 0.89 | 14.31 |
| rs13015552 | T | C | -0.0029 | -0.001412 | 0.3806 | 0.38128 | 5.00E-04 | 6.78E-10 | 0.3 | 15.86 |
| rs13039514 | T | G | 0.0628 | 0.0001598 | 0.0205 | 0.021061 | 0.0017 | 1E-200 | 0.97 | 54.81 |
| rs1317983 | T | C | 0.0067 | -0.000905 | 0.3075 | 0.305975 | 5.00E-04 | 1.38E-41 | 0.53 | 76.48 |
| rs13230509 | C | G | -0.0068 | -0.000373 | 0.6862 | 0.691899 | 5.00E-04 | 1.63E-39 | 0.8 | 79.67 |
| rs13329240 | A | G | -0.0036 | -2.88E-04 | 0.3193 | 0.317623 | 5.00E-04 | 2.12E-13 | 0.84 | 22.54 |
| rs147988763 | C | G | -0.0552 | 0.0021749 | 0.9631 | 0.962233 | 1.20E-03 | 1.00E-200 | 0.54 | 150.45 |
| rs151245 | T | G | -0.0029 | 0.0002452 | 0.5996 | 0.600734 | 5.00E-04 | 1.05E-09 | 0.86 | 16.15 |
| rs1585499 | T | C | -0.0046 | 0.000294 | 0.4552 | 0.461402 | 5.00E-04 | 1.40E-23 | 0.83 | 41.98 |
| rs16956623 | T | C | 0.0035 | -0.003231 | 0.2883 | 0.281194 | 5.00E-04 | 7.08E-12 | 0.028 | 20.11 |
| rs17420882 | T | G | 0.0035 | 0.001147 | 0.7238 | 0.720866 | 5.00E-04 | 1.16E-11 | 0.43 | 19.59 |
| rs174541 | T | C | -0.0028 | -0.00103 | 0.6414 | 0.638589 | 5.00E-04 | 2.79E-09 | 0.45 | 14.43 |
| rs17693971 | C | G | 0.0045 | -0.003117 | 0.6856 | 0.681419 | 0.0005 | 1.16E-19 | 0.028 | 34.92 |
| rs198325 | T | C | 0.0053 | -0.00027 | 0.2155 | 0.221296 | 6.00E-04 | 9.05E-22 | 0.86 | 26.38 |
| rs2014520 | A | G | 0.0031 | 0.0004464 | 0.4113 | 0.421699 | 5.00E-04 | 3.43E-11 | 0.74 | 18.62 |
| rs2049019 | A | C | 0.0034 | -0.00165 | 0.6806 | 0.686382 | 5.00E-04 | 9.81E-12 | 0.25 | 20.10 |
| rs2068888 | A | G | -0.0036 | -0.004529 | 0.4524 | 0.447761 | 5.00E-04 | 1.45E-14 | 0.00063 | 25.69 |
| rs2104032 | C | G | -0.0027 | 0.0020214 | 0.4038 | 0.402025 | 5.00E-04 | 1.30E-08 | 0.13 | 14.04 |
| rs2151421 | A | G | 0.0043 | 0.0019879 | 0.6464 | 0.641892 | 5.00E-04 | 4.55E-19 | 0.15 | 33.81 |
| rs2161796 | T | C | -0.0026 | 0.0008372 | 0.6222 | 0.624635 | 5.00E-04 | 4.68E-08 | 0.54 | 12.71 |
| rs219778 | A | G | -0.0034 | 0.0012849 | 0.7372 | 0.73586 | 5.00E-04 | 4.28E-11 | 0.39 | 17.92 |
| rs2213732 | A | G | -0.0028 | -1.85E-03 | 0.5673 | 0.56826 | 5.00E-04 | 2.26E-09 | 0.17 | 15.40 |
| rs223485 | A | T | 0.0042 | -0.001956 | 0.4884 | 0.489452 | 5.00E-04 | 5.29E-20 | 0.14 | 35.26 |
| rs2241358 | C | G | -0.0027 | 0.0037971 | 0.6724 | 0.669957 | 5.00E-04 | 3.53E-08 | 0.0069 | 12.85 |
| rs224146 | A | G | -0.0033 | -0.002473 | 0.4014 | 0.397548 | 5.00E-04 | 2.64E-12 | 0.067 | 20.93 |
| rs2282718 | A | G | 0.0027 | -0.001884 | 0.3736 | 0.371224 | 5.00E-04 | 1.70E-08 | 0.17 | 13.65 |
| rs2290846 | A | G | -0.003 | 0.0028406 | 0.283 | 0.289849 | 5.00E-04 | 4.96E-09 | 0.051 | 14.61 |
| rs2306623 | T | C | -0.0034 | 0.0015762 | 0.3333 | 0.33324 | 5.00E-04 | 5.78E-12 | 0.26 | 20.55 |
| rs2307111 | T | C | -0.0028 | -0.001136 | 0.598 | 0.606863 | 5.00E-04 | 1.33E-09 | 0.4 | 15.08 |
| rs241437 | A | G | 0.0028 | 0.0019528 | 0.6046 | 0.605045 | 5.00E-04 | 2.57E-09 | 0.15 | 14.99 |
| rs2444820 | C | G | -0.003 | -0.000708 | 0.4334 | 0.434648 | 5.00E-04 | 5.99E-11 | 0.6 | 17.68 |
| rs2464196 | A | G | -0.0058 | -0.000114 | 0.2952 | 0.291256 | 5.00E-04 | 9.57E-31 | 0.94 | 56.00 |
| rs2542713 | A | C | -0.0037 | -8.12E-04 | 0.4266 | 0.422293 | 5.00E-04 | 1.72E-15 | 0.54 | 26.79 |
| rs2638281 | A | G | 0.0027 | 6.786E-05 | 0.405 | 0.398056 | 5.00E-04 | 1.69E-08 | 0.96 | 14.05 |
| rs2755253 | T | C | -0.0043 | -0.000417 | 0.7081 | 0.706566 | 5.00E-04 | 1.73E-17 | 0.77 | 30.58 |
| rs2823139 | A | G | -0.0038 | -0.000273 | 0.3369 | 0.33838 | 5.00E-04 | 7.46E-15 | 0.85 | 25.81 |
| rs2834322 | A | G | -0.0053 | 0.0015036 | 0.1797 | 0.180815 | 6.00E-04 | 9.89E-19 | 0.38 | 23.00 |
| rs28394165 | T | C | 0.0115 | 0.0031056 | 0.5522 | 0.541685 | 5.00E-04 | 5.24E-137 | 0.019 | 261.76 |
| rs288762 | T | C | 0.005 | 0.0004035 | 0.3639 | 0.362989 | 5.00E-04 | 2.36E-25 | 0.77 | 46.30 |
| rs28930677 | T | C | -0.0118 | -0.002103 | 0.0462 | 0.046847 | 1.10E-03 | 1.42E-26 | 0.5 | 10.14 |
| rs2969473 | C | G | 0.0032 | 0.0001217 | 0.3177 | 0.325223 | 5.00E-04 | 1.46E-10 | 0.93 | 17.76 |
| rs303940 | A | G | 0.0045 | 0.002196 | 0.4043 | 0.393266 | 0.0005 | 1.41E-21 | 0.1 | 39.02 |
| rs310757 | T | C | 0.0028 | -1.03E-03 | 0.4602 | 0.458231 | 5.00E-04 | 1.14E-09 | 0.44 | 15.58 |
| rs3184504 | T | C | -0.0108 | 0.0020525 | 0.4726 | 0.482707 | 5.00E-04 | 1.02E-122 | 0.12 | 232.70 |
| rs3197999 | A | G | 0.0035 | -0.000247 | 0.2832 | 0.28397 | 5.00E-04 | 1.22E-11 | 0.87 | 19.89 |
| rs33062 | T | C | -0.0063 | 0.0036178 | 0.1503 | 0.153688 | 6.00E-04 | 1.10E-22 | 0.049 | 28.16 |
| rs332129 | A | G | 0.0028 | 0.0010141 | 0.5235 | 0.522462 | 5.00E-04 | 5.74E-10 | 0.44 | 15.65 |
| rs333947 | A | G | -0.0058 | -0.001878 | 0.151 | 0.149748 | 6.00E-04 | 1.05E-19 | 0.31 | 23.96 |
| rs34046597 | T | C | -0.0028 | -0.00043 | 0.5456 | 0.545626 | 5.00E-04 | 8.92E-10 | 0.75 | 15.55 |
| rs34517439 | A | C | -0.0065 | 0.0020386 | 0.1196 | 0.124422 | 7.00E-04 | 7.00E-20 | 0.31 | 18.16 |
| rs35325993 | T | C | -0.0033 | -0.000912 | 0.2997 | 0.301998 | 5.00E-04 | 4.55E-11 | 0.53 | 18.29 |
| rs35490232 | T | C | 0.0029 | 0.0013605 | 0.5144 | 0.518319 | 5.00E-04 | 4.55E-10 | 0.3 | 16.81 |
| rs35506085 | A | G | 0.0052 | -0.002251 | 0.1868 | 0.182711 | 6.00E-04 | 3.44E-18 | 0.19 | 22.82 |
| rs35646918 | A | G | -0.0048 | -0.001094 | 0.1723 | 0.175377 | 6.00E-04 | 2.00E-15 | 0.53 | 18.26 |
| rs357503 | A | G | 0.0029 | 0.0018198 | 0.2855 | 0.284471 | 5.00E-04 | 1.00E-08 | 0.21 | 13.72 |
| rs3757387 | T | C | 0.0063 | -0.002495 | 0.552 | 0.546235 | 5.00E-04 | 2.40E-42 | 0.06 | 78.53 |
| rs3762727 | T | G | 0.0031 | -0.003209 | 0.2862 | 0.284367 | 5.00E-04 | 1.65E-09 | 0.029 | 15.71 |
| rs3790585 | A | T | 0.0046 | -0.006388 | 0.8405 | 0.841619 | 6.00E-04 | 4.35E-13 | 0.00042 | 15.76 |
| rs3925584 | T | C | -0.0068 | 0.0010662 | 0.5499 | 0.544461 | 5.00E-04 | 8.12E-49 | 0.42 | 91.58 |
| rs41276588 | A | G | -0.0031 | -0.00042 | 0.2802 | 0.28684 | 5.00E-04 | 9.72E-10 | 0.77 | 15.51 |
| rs4149056 | T | C | -0.004 | 0.0009213 | 0.8498 | 0.849626 | 6.00E-04 | 4.77E-10 | 0.62 | 11.35 |
| rs429358 | T | C | -0.0056 | -0.00173 | 0.8456 | 0.84458 | 6.00E-04 | 2.88E-18 | 0.34 | 22.75 |
| rs4442348 | A | G | 0.0028 | 0.0027537 | 0.5016 | 0.500111 | 5.00E-04 | 7.63E-10 | 0.037 | 15.68 |
| rs45604939 | A | G | -0.0108 | -0.000845 | 0.9315 | 0.930123 | 9.00E-04 | 6.54E-33 | 0.74 | 18.38 |
| rs4589131 | T | G | -0.0027 | 0.0026146 | 0.4053 | 0.409583 | 5.00E-04 | 5.72E-09 | 0.051 | 14.06 |
| rs4672892 | A | G | -0.0042 | -0.000414 | 0.6646 | 0.657733 | 5.00E-04 | 5.12E-18 | 0.77 | 31.46 |
| rs4715317 | T | G | 0.0031 | 0.0004712 | 0.3511 | 0.354182 | 5.00E-04 | 1.63E-10 | 0.73 | 17.52 |
| rs4959168 | T | C | 0.0031 | 0.0007274 | 0.6305 | 0.626374 | 5.00E-04 | 6.93E-11 | 0.59 | 17.91 |
| rs4966019 | T | C | 0.0061 | -0.002402 | 0.6356 | 0.641074 | 5.00E-04 | 1.51E-37 | 0.081 | 68.96 |
| rs4973560 | T | C | -0.0029 | 0.0019459 | 0.5847 | 0.582084 | 5.00E-04 | 7.98E-10 | 0.15 | 16.34 |
| rs498010 | T | C | 0.0036 | 0.0003751 | 0.1872 | 0.18303 | 6.00E-04 | 2.21E-09 | 0.83 | 10.96 |
| rs55753056 | A | G | -0.0041 | -0.001784 | 0.3452 | 0.350469 | 5.00E-04 | 4.21E-17 | 0.2 | 30.40 |
| rs56144347 | T | C | 0.0039 | -0.002382 | 0.2833 | 0.281327 | 5.00E-04 | 3.54E-14 | 0.1 | 24.71 |
| rs571717 | T | C | -0.0026 | 0.0020448 | 0.3837 | 0.387469 | 5.00E-04 | 2.20E-08 | 0.13 | 12.79 |
| rs6026578 | C | G | -0.0034 | -0.000577 | 0.3755 | 0.375259 | 5.00E-04 | 2.07E-12 | 0.67 | 21.69 |
| rs6127099 | A | T | -0.006 | 0.0008579 | 0.7188 | 0.721476 | 5.00E-04 | 2.00E-30 | 0.57 | 58.22 |
| rs621634 | T | G | 0.0034 | 0.0015747 | 0.258 | 0.261823 | 5.00E-04 | 9.25E-11 | 0.3 | 17.70 |
| rs62483619 | T | C | 0.0039 | 0.0016101 | 0.2357 | 0.235438 | 5.00E-04 | 4.81E-13 | 0.3 | 21.92 |
| rs6429746 | A | G | -0.003 | 0.0015922 | 0.3167 | 0.315111 | 5.00E-04 | 1.67E-09 | 0.26 | 15.58 |
| rs6433958 | T | C | -0.0027 | -9.88E-05 | 0.4891 | 0.489375 | 5.00E-04 | 3.00E-09 | 0.94 | 14.57 |
| rs6445528 | A | G | -0.0025 | 0.0008969 | 0.5258 | 0.527504 | 5.00E-04 | 3.32E-08 | 0.5 | 12.47 |
| rs6460047 | T | C | 0.0039 | -0.004557 | 0.7932 | 0.791246 | 6.00E-04 | 5.40E-12 | 0.0051 | 13.86 |
| rs6567160 | T | C | 0.0034 | 0.0006393 | 0.7684 | 0.766002 | 5.00E-04 | 2.40E-10 | 0.68 | 16.46 |
| rs6715105 | T | C | 0.0031 | -0.00068 | 0.3253 | 0.326167 | 5.00E-04 | 3.15E-10 | 0.63 | 16.87 |
| rs6787409 | T | C | -0.0044 | 0.0001444 | 0.6766 | 0.670943 | 5.00E-04 | 1.12E-18 | 0.92 | 33.89 |
| rs68147365 | A | G | 0.0047 | 0.0001209 | 0.237 | 0.243765 | 5.00E-04 | 2.15E-18 | 0.94 | 31.96 |
| rs6873866 | T | C | 0.0027 | -0.002384 | 0.4337 | 0.437575 | 5.00E-04 | 7.35E-09 | 0.073 | 14.32 |
| rs687467 | A | G | 0.0053 | -0.001961 | 0.2532 | 0.242149 | 5.00E-04 | 6.52E-23 | 0.21 | 42.50 |
| rs687914 | T | G | -0.0036 | -0.001201 | 0.2468 | 0.251958 | 5.00E-04 | 2.10E-11 | 0.43 | 19.27 |
| rs6999484 | A | G | -0.0074 | 0.0034078 | 0.4082 | 0.412719 | 5.00E-04 | 7.99E-57 | 0.011 | 105.85 |
| rs700750 | A | C | -0.0045 | -0.000435 | 0.63 | 0.628944 | 5.00E-04 | 1.64E-21 | 0.75 | 37.77 |
| rs7123220 | T | C | 0.0034 | 0.0009891 | 0.3849 | 0.38488 | 5.00E-04 | 7.21E-13 | 0.47 | 21.90 |
| rs71359461 | C | G | -0.0039 | 0.0014476 | 0.4791 | 0.481163 | 5.00E-04 | 8.25E-17 | 0.28 | 30.37 |
| rs7140970 | A | G | -0.0026 | -0.001387 | 0.5779 | 0.570372 | 5.00E-04 | 4.60E-08 | 0.32 | 13.19 |
| rs7206629 | T | C | 0.0041 | 0.0075817 | 0.5877 | 0.586635 | 5.00E-04 | 9.54E-19 | 1.5E-08 | 32.59 |
| rs726311 | C | G | -0.0028 | 0.0010407 | 0.6028 | 0.598036 | 5.00E-04 | 3.36E-09 | 0.44 | 15.02 |
| rs72759874 | C | G | 0.0055 | -0.001903 | 0.8951 | 0.892023 | 7.00E-04 | 7.48E-14 | 0.37 | 11.59 |
| rs728538 | T | G | 0.0043 | -0.004252 | 0.8322 | 0.832835 | 6.00E-04 | 3.65E-12 | 0.017 | 14.34 |
| rs72946069 | T | C | -0.0035 | -0.000224 | 0.8118 | 0.810109 | 0.0006 | 1.99E-09 | 0.89 | 10.40 |
| rs73312653 | A | G | -0.0208 | -0.000138 | 0.1714 | 0.16979 | 6.00E-04 | 1.00E-200 | 0.94 | 341.61 |
| rs7459014 | A | C | 0.0031 | -0.000537 | 0.2744 | 0.276279 | 5.00E-04 | 5.62E-09 | 0.72 | 15.31 |
| rs7502669 | A | G | -0.0027 | 0.0023791 | 0.4174 | 0.410284 | 5.00E-04 | 1.50E-08 | 0.079 | 14.18 |
| rs7535162 | T | G | 0.0033 | 0.0004385 | 0.6802 | 0.678146 | 5.00E-04 | 1.62E-11 | 0.76 | 18.95 |
| rs7623754 | A | G | -0.0037 | -0.002953 | 0.4753 | 0.480686 | 5.00E-04 | 1.06E-15 | 0.025 | 27.31 |
| rs76512888 | T | C | -0.0051 | 0.0029502 | 0.863 | 0.860184 | 7.00E-04 | 2.45E-14 | 0.12 | 12.55 |
| rs76798800 | T | G | -0.0044 | -0.005758 | 0.2629 | 0.26653 | 5.00E-04 | 1.75E-17 | 0.00012 | 30.02 |
| rs7684939 | A | G | 0.0033 | 0.0003198 | 0.4851 | 0.490451 | 5.00E-04 | 3.44E-13 | 0.81 | 21.76 |
| rs76862947 | T | C | 0.0037 | 0.0001449 | 0.7114 | 0.702906 | 5.00E-04 | 2.25E-13 | 0.92 | 22.49 |
| rs7708715 | A | C | 0.0025 | -0.002084 | 0.4713 | 0.464926 | 5.00E-04 | 3.59E-08 | 0.12 | 12.46 |
| rs7786368 | T | C | 0.0041 | 0.001069 | 0.5852 | 0.58309 | 5.00E-04 | 1.89E-18 | 0.42 | 32.65 |
| rs77924615 | A | G | 0.012 | 0.0008122 | 0.1958 | 0.197991 | 6.00E-04 | 6.29E-94 | 0.63 | 126.00 |
| rs79103983 | T | G | -0.0036 | -0.002968 | 0.8215 | 0.819461 | 6.00E-04 | 2.24E-09 | 0.085 | 10.56 |
| rs79251791 | T | C | 0.0072 | 0.0014545 | 0.9179 | 0.919198 | 8.00E-04 | 1.02E-17 | 0.55 | 12.21 |
| rs7944897 | A | G | 0.0032 | 0.0007613 | 0.5415 | 0.536025 | 5.00E-04 | 4.16E-12 | 0.56 | 20.34 |
| rs80182235 | A | G | 0.0208 | 0.000147 | 0.0282 | 0.028239 | 1.40E-03 | 1.44E-50 | 0.97 | 12.10 |
| rs80282103 | A | T | 0.0087 | 0.0015132 | 0.915 | 0.916175 | 8.00E-04 | 2.38E-25 | 0.53 | 18.40 |
| rs807624 | T | G | 0.0054 | -0.001923 | 0.3663 | 0.358391 | 5.00E-04 | 4.74E-29 | 1.60E-01 | 54.16 |
| rs8101667 | T | C | 0.005 | -0.000324 | 0.3418 | 0.339123 | 5.00E-04 | 3.56E-25 | 0.82 | 45.00 |
| rs836968 | T | C | 0.004 | -0.001431 | 0.2752 | 0.270185 | 5.00E-04 | 1.47E-14 | 0.34 | 25.53 |
| rs849089 | A | G | 0.0031 | -0.001334 | 0.319 | 0.317742 | 0.0005 | 5.448E-10 | 0.35 | 16.70 |
| rs863678 | T | G | -0.0049 | -0.000594 | 0.6423 | 0.642227 | 0.0005 | 2.273E-23 | 0.67 | 44.13 |
| rs9272657 | A | G | -0.0077 | -0.006353 | 0.1796 | 0.178087 | 0.0007 | 1.975E-30 | 0.00097 | 35.66 |
| rs9307594 | A | G | 0.0028 | 0.0016234 | 0.5306 | 0.536371 | 0.0005 | 8.849E-10 | 0.22 | 15.62 |
| rs9361858 | T | G | 0.0049 | 0.0021176 | 0.5772 | 0.582295 | 0.0005 | 2.248E-25 | 0.12 | 46.88 |
| rs9465741 | A | C | 0.0027 | 0.000686 | 0.5408 | 0.550772 | 0.0005 | 5.796E-09 | 0.61 | 14.48 |
| rs9534949 | C | G | -0.0029 | 0.001649 | 0.277 | 0.270229 | 0.0005 | 2.216E-08 | 0.27 | 13.47 |
| rs9838515 | C | G | 0.003 | -0.002423 | 0.2566 | 0.25401 | 0.0005 | 1.415E-08 | 0.11 | 13.73 |
| rs9905543 | T | C | 0.0061 | 0.0054898 | 0.762 | 0.762747 | 0.0005 | 3.08E-30 | 0.00039 | 53.99 |
| rs9930127 | A | G | 0.0032 | -0.000897 | 0.4743 | 0.474204 | 0.0005 | 4.286E-12 | 0.5 | 20.43 |
| rs9930360 | T | C | -0.0033 | 0.0010295 | 0.7626 | 0.765459 | 0.0005 | 7.397E-10 | 0.51 | 15.77 |

11 SNPs (rs10010367, rs1260326, rs2464196, rs2638281, rs3184504, rs429358, rs56144347, rs7206629, rs76798800, rs7684939, rs77924615) that were removed from Pheno Scanner step, and 5 SNPs (rs10782784; rs10824218; rs223485; rs2444820; rs71359461) that were removed from harmonization step.

**Table S6** Effect estimates for the associations of the genetic variants with UACR and hearing loss.

| **SNP** | **effect_allele** | **other_allele** | **beta.exposure** | **beta.outcome** | **eaf.exposure** | **eaf.outcome** | **se.exposure** | **pval.exposure** | **se.outcome** | **pval.outcome** | **F_statisic** |
| --- | --- | --- | --- | --- | --- | --- | --- | --- | --- | --- | --- |
| rs10023335 | T | C | 0.01439 | 0.003236 | 0.5932 | 0.589601 | 0.002017 | 9.72E-13 | 0 | 0.016 | 24.57 |
| rs10207567 | C | G | 0.0193712 | 0.0025249 | 0.8151 | 0.811933 | 0.002552 | 3.18E-14 | 0 | 0.13 | 17.37 |
| rs1047891 | A | C | -0.018991 | 0.001028 | 0.3148 | 0.314648 | 0.0021754 | 2.55E-18 | 0 | 0.47 | 32.88 |
| rs1057868 | T | C | 0.0121717 | -0.003522 | 0.2846 | 0.285931 | 0.0021985 | 3.09E-08 | 0 | 0.016 | 12.48 |
| rs1086899 | T | C | -0.01237 | 0.0025881 | 0.2903 | 0.286797 | 0.0022318 | 2.98E-08 | 0 | 0.076 | 12.66 |
| rs11078597 | T | C | -0.015992 | -0.002164 | 0.813 | 0.814361 | 0.0025948 | 7.13E-10 | 0 | 0.2 | 11.55 |
| rs11709284 | A | G | 0.0112451 | -0.000555 | 0.5589 | 0.555327 | 0.0019991 | 1.86E-08 | 0 | 0.68 | 15.60 |
| rs11912350 | T | C | -0.013007 | 0.0021854 | 0.7582 | 0.75861 | 0.002332 | 2.44E-08 | 0 | 0.16 | 11.41 |
| rs12714144 | A | T | 0.0224637 | -0.000616 | 0.8733 | 0.872574 | 0.0029873 | 5.49E-14 | 0 | 0.76 | 12.51 |
| rs12790943 | T | C | 0.0136538 | 0.0013562 | 0.422 | 0.421575 | 0.0020126 | 1.17E-11 | 0 | 0.31 | 22.45 |
| rs1309546 | T | C | 0.0123644 | 0.000234 | 0.5515 | 0.550753 | 0.0019965 | 5.90E-10 | 0 | 0.86 | 18.97 |
| rs13132085 | A | G | -0.012762 | -0.000693 | 0.2893 | 0.288999 | 0.0021969 | 6.28E-09 | 0 | 0.64 | 13.88 |
| rs1337526 | A | G | -0.027088 | 0.0007214 | 0.1983 | 0.199162 | 0.0024882 | 1.34E-27 | 0 | 0.66 | 37.69 |
| rs15052 | T | C | 0.0173241 | 0.0002562 | 0.8253 | 0.821387 | 0.0027287 | 2.17E-10 | 0 | 0.88 | 11.62 |
| rs162890 | T | C | 0.0134553 | 0.0007062 | 0.3317 | 0.331789 | 0.002176 | 6.27E-10 | 0 | 0.62 | 16.95 |
| rs1688031 | T | C | -0.019485 | 0.0006102 | 0.142 | 0.13906 | 0.002893 | 1.64E-11 | 0 | 0.75 | 11.05 |
| rs17035646 | A | G | 0.0120302 | -0.000981 | 0.3398 | 0.337828 | 0.0021185 | 1.36E-08 | 0 | 0.48 | 14.47 |
| rs17158386 | A | G | 0.019817 | -0.000663 | 0.2584 | 0.260852 | 0.0023357 | 2.17E-17 | 0 | 0.66 | 27.59 |
| rs17343073 | A | T | -0.061987 | -0.002758 | 0.8958 | 0.896965 | 0.003253 | 5.94E-81 | 0 | 0.2 | 67.80 |
| rs2068888 | A | G | -0.012427 | -0.004529 | 0.4515 | 0.447761 | 0.0020029 | 5.50E-10 | 0 | 0.00063 | 19.07 |
| rs2277537 | A | G | 0.0149652 | 0.0006538 | 0.597 | 0.598953 | 0.0020316 | 1.76E-13 | 0 | 0.63 | 26.11 |
| rs2433611 | A | C | -0.017544 | 0.0034228 | 0.2594 | 0.256466 | 0.0022635 | 9.11E-15 | 0 | 0.023 | 23.08 |
| rs2470893 | T | C | 0.0230342 | 0.0019342 | 0.3259 | 0.332546 | 0.0021538 | 1.08E-26 | 0 | 0.17 | 50.26 |
| rs2601006 | T | C | -0.015452 | -0.000578 | 0.343 | 0.34204 | 0.0020932 | 1.56E-13 | 0 | 0.68 | 24.56 |
| rs2954021 | A | G | 0.0148457 | -0.000141 | 0.4915 | 0.493123 | 0.0019821 | 6.89E-14 | 0 | 0.92 | 28.04 |
| rs34257409 | T | G | 0.0160895 | 0.0002825 | 0.4038 | 0.405462 | 0.0020197 | 1.63E-15 | 0 | 0.83 | 30.56 |
| rs35572189 | A | G | -0.011987 | -0.003143 | 0.3641 | 0.361778 | 0.0021641 | 3.05E-08 | 0 | 0.022 | 14.21 |
| rs35692677 | A | G | -0.016352 | -0.000412 | 0.1863 | 0.187738 | 0.0025946 | 2.93E-10 | 0 | 0.81 | 12.04 |
| rs3734692 | A | T | -0.017723 | 0.0004861 | 0.6908 | 0.68974 | 0.0021814 | 4.49E-16 | 0 | 0.73 | 28.20 |
| rs4410790 | T | C | -0.021924 | -0.000439 | 0.3689 | 0.365035 | 0.0020613 | 2.03E-26 | 0 | 0.75 | 52.68 |
| rs4641276 | T | C | -0.012841 | -0.002032 | 0.2455 | 0.246913 | 0.0023308 | 3.61E-08 | 0 | 0.19 | 11.24 |
| rs4665972 | T | C | 0.0173726 | 0.0017459 | 0.3953 | 0.392593 | 0.0020777 | 6.20E-17 | 0 | 0.2 | 33.43 |
| rs4738817 | A | G | -0.011531 | -0.002614 | 0.4535 | 0.451176 | 0.0019942 | 7.39E-09 | 0 | 0.049 | 16.57 |
| rs4899263 | A | G | -0.013624 | -0.00301 | 0.5302 | 0.52956 | 0.0020211 | 1.57E-11 | 0 | 0.023 | 22.64 |
| rs56336142 | T | C | -0.017307 | -0.000988 | 0.7876 | 0.787141 | 0.002439 | 1.29E-12 | 0 | 0.54 | 16.85 |
| rs6119771 | C | G | 0.0112904 | 0.0013896 | 0.4257 | 0.423269 | 0.0020089 | 1.91E-08 | 0 | 0.3 | 15.44 |
| rs6535594 | A | G | 0.0143694 | 0.0032918 | 0.4979 | 0.494806 | 0.0019939 | 5.74E-13 | 0 | 0.013 | 25.97 |
| rs67339103 | A | G | 0.0172135 | 0.001317 | 0.2163 | 0.212254 | 0.0024569 | 2.45E-12 | 0 | 0.42 | 16.64 |
| rs677888 | T | G | -0.014319 | -0.001011 | 0.7598 | 0.759022 | 0.0023247 | 7.30E-10 | 0 | 0.51 | 13.85 |
| rs7115200 | T | G | -0.012255 | -0.000599 | 0.5605 | 0.560078 | 0.0020438 | 2.02E-09 | 0 | 0.65 | 17.71 |
| rs7812843 | A | G | -0.0117 | 0.0016964 | 0.501 | 0.499222 | 0.0019827 | 3.62E-09 | 0 | 0.2 | 17.41 |
| rs838142 | A | G | 0.017184 | -0.001597 | 0.7203 | 0.722348 | 0.0023041 | 8.78E-14 | 0 | 0.28 | 22.41 |
| rs988712 | T | G | -0.013175 | -0.003875 | 0.2363 | 0.235163 | 0.0023395 | 1.79E-08 | 0 | 0.013 | 11.45 |

One SNP (rs6119771) that were removed from harmonization step.

**Table S7** Effect estimates for the associations of the genetic variants with urate and hearing loss.

| **SNP** | **effect_allele** | **other_allele** | **beta.exposure** | **beta.outcome** | **eaf.exposure** | **eaf.outcome** | **se.exposure** | **pval.exposure** | **se.outcome** | **pval.outcome** | **F_statisic** |
| --- | --- | --- | --- | --- | --- | --- | --- | --- | --- | --- | --- |
| rs10223666 | C | G | 0.046445 | 0.0009558 | 0.7036 | 0.694228 | 0.0042414 | 6.62E-28 | 0.0014348 | 0.51 | 50.02 |
| rs10405423 | A | C | 0.03865 | 0.0003532 | 0.6625 | 0.658356 | 0.004143 | 1.07E-20 | 0.0014063 | 0.8 | 38.92 |
| rs1047891 | A | C | -0.023735 | 0.001028 | 0.3107 | 0.314648 | 0.0042351 | 2.09E-08 | 0.0014199 | 0.47 | 13.45 |
| rs10480300 | T | C | 0.030123 | -0.003763 | 0.2755 | 0.273787 | 0.0043479 | 4.26E-12 | 0.0014799 | 0.011 | 19.16 |
| rs10774625 | A | G | 0.032335 | 0.0021877 | 0.4826 | 0.496365 | 0.003861 | 5.54E-17 | 0.0013192 | 0.097 | 35.03 |
| rs10851885 | A | G | -0.05396 | 0.0008153 | 0.7559 | 0.742278 | 0.004575 | 4.16E-32 | 0.0015266 | 0.59 | 51.34 |
| rs10892354 | T | C | 0.030101 | 0.0002331 | 0.38 | 0.39563 | 0.0041158 | 2.60E-13 | 0.0013652 | 0.86 | 25.21 |
| rs10910845 | A | C | 0.057964 | -0.000283 | 0.4692 | 0.462763 | 0.0038374 | 1.50E-51 | 0.0013229 | 0.83 | 113.69 |
| rs10942549 | C | G | -0.042149 | -0.003502 | 0.312 | 0.313448 | 0.0043177 | 1.64E-22 | 0.0014286 | 0.014 | 40.92 |
| rs10956924 | T | C | -0.023868 | -0.003788 | 0.2793 | 0.268871 | 0.0042382 | 1.79E-08 | 0.0014884 | 0.011 | 12.77 |
| rs10971420 | T | C | 0.030745 | 9.498E-05 | 0.6875 | 0.687162 | 0.0040688 | 4.14E-14 | 0.0014241 | 0.95 | 24.54 |
| rs10994731 | A | G | -0.060861 | -2.44E-03 | 0.8816 | 0.877525 | 0.0061452 | 4.01E-23 | 0.0020224 | 0.23 | 20.48 |
| rs11128111 | T | C | -0.020891 | 0.0028026 | 0.48 | 0.479795 | 0.003823 | 4.64E-08 | 0.0013319 | 0.035 | 14.91 |
| rs111625726 | T | C | 0.075871 | -0.000434 | 0.7015 | 0.694842 | 0.0042661 | 9.33E-71 | 0.0014444 | 0.76 | 132.52 |
| rs114750851 | A | G | 0.269772 | -0.005569 | 0.9687 | 0.969748 | 0.0122554 | 2.19E-107 | 0.0038969 | 0.15 | 29.39 |
| rs11551890 | A | G | 0.02308 | 0.0018685 | 0.5087 | 0.48869 | 0.0041358 | 2.40E-08 | 0.0013638 | 0.17 | 15.57 |
| rs11614136 | A | T | -0.029063 | -1.22E-03 | 0.6439 | 0.639695 | 0.0040751 | 9.90E-13 | 0.0013795 | 0.38 | 23.33 |
| rs11663816 | T | C | -0.030377 | 6.755E-05 | 0.7295 | 0.732188 | 0.0042881 | 1.40E-12 | 0.0014918 | 0.96 | 19.81 |
| rs1171617 | T | G | 0.079207 | 0.000229 | 0.7682 | 0.767644 | 0.0045991 | 1.81E-66 | 0.0015636 | 0.88 | 105.67 |
| rs117864137 | A | G | 0.028299 | 0.002052 | 0.731 | 0.747437 | 0.0043041 | 4.87E-11 | 0.0015243 | 0.18 | 17.00 |
| rs12037861 | A | T | 0.023078 | 0.0025878 | 0.7041 | 0.705218 | 0.0041809 | 3.39E-08 | 0.0014481 | 0.074 | 12.70 |
| rs12313306 | T | C | -0.076333 | 0.0001804 | 0.2464 | 0.244093 | 0.0044871 | 6.74E-65 | 0.0015343 | 0.91 | 107.51 |
| rs1234413 | T | C | -0.022345 | 0.0012062 | 0.4415 | 0.452899 | 0.0038599 | 7.08E-09 | 0.0013272 | 0.36 | 16.53 |
| rs12423664 | A | G | 0.041927 | -0.000469 | 0.1515 | 0.14021 | 0.0056912 | 1.75E-13 | 0.0019002 | 0.8 | 13.95 |
| rs12472381 | A | G | 0.021931 | 0.0016548 | 0.39 | 0.398175 | 0.0038952 | 1.80E-08 | 0.0013488 | 0.22 | 15.08 |
| rs12485100 | T | G | -0.03262 | -0.001583 | 0.1726 | 0.157352 | 0.0051526 | 2.44E-10 | 0.0018097 | 0.38 | 11.45 |
| rs12530084 | T | C | 0.06643 | -0.001063 | 0.2198 | 0.230927 | 0.0045762 | 9.55E-48 | 0.0015663 | 0.5 | 72.29 |
| rs12576996 | T | G | -0.046872 | -0.002744 | 0.7372 | 0.754293 | 0.0044309 | 3.75E-26 | 0.001542 | 0.075 | 43.37 |
| rs1260326 | T | C | 0.069602 | 0.0020343 | 0.3983 | 0.392533 | 0.0039626 | 4.61E-69 | 0.0013482 | 0.13 | 147.95 |
| rs12908437 | T | C | 0.045764 | 0.0025422 | 0.3761 | 0.364069 | 0.0039845 | 1.56E-30 | 0.0013757 | 0.065 | 61.92 |
| rs12987661 | T | C | 0.041324 | 0.0004658 | 0.8657 | 0.872375 | 0.0058364 | 1.44E-12 | 0.0019739 | 0.81 | 11.66 |
| rs13226650 | A | G | 0.048744 | -5.22E-03 | 0.8085 | 0.800525 | 0.0048683 | 1.35E-23 | 0.001651 | 0.0016 | 31.05 |
| rs1359232 | A | C | -0.090844 | -0.000886 | 0.466 | 0.432543 | 0.003803 | 4.12E-126 | 0.001331 | 0.51 | 284.26 |
| rs1383585 | A | G | -0.202781 | 0.0004587 | 0.9018 | 0.901716 | 0.0064003 | 1E-200 | 0.0022107 | 0.84 | 177.90 |
| rs1440411 | T | C | -0.027552 | 8.558E-05 | 0.5706 | 0.574789 | 0.0038698 | 1.08E-12 | 0.0013389 | 0.95 | 24.84 |
| rs1478604 | T | C | -0.026249 | 0.0007015 | 0.7061 | 0.703424 | 0.0042092 | 4.49E-10 | 0.0014497 | 0.63 | 16.14 |
| rs16889260 | T | C | -0.154484 | 0.003047 | 0.1857 | 0.187225 | 0.0049255 | 1.00E-200 | 0.001696 | 0.072 | 297.81 |
| rs16892069 | T | C | 0.188963 | -0.003208 | 0.9463 | 0.955716 | 0.0090334 | 3.65E-97 | 0.0032299 | 0.32 | 44.48 |
| rs17050272 | A | G | 0.031806 | 0.0008955 | 0.421 | 0.411021 | 0.00399 | 1.57E-15 | 0.0013412 | 0.5 | 30.98 |
| rs2070803 | A | G | 0.052586 | -0.002213 | 0.5777 | 0.573228 | 0.0039158 | 4.09E-41 | 0.001332 | 0.097 | 88.02 |
| rs2108878 | T | C | 0.107314 | 0.0004223 | 0.7033 | 0.713116 | 0.0042171 | 7.54E-143 | 0.0014602 | 0.77 | 270.51 |
| rs219781 | T | G | -0.025151 | -0.001387 | 0.2456 | 0.263666 | 0.0044479 | 1.56E-08 | 0.0014977 | 0.35 | 11.85 |
| rs2453580 | T | C | 0.024703 | -0.00203 | 0.5977 | 0.611996 | 0.0040064 | 7.01E-10 | 0.0013568 | 0.13 | 18.28 |
| rs2480712 | C | G | 0.024325 | 0.0020194 | 0.6621 | 0.661842 | 0.0041868 | 6.25E-09 | 0.0014025 | 0.15 | 15.10 |
| rs2581817 | C | G | 0.047911 | 0.0017765 | 0.4198 | 0.423931 | 0.0038795 | 4.87E-35 | 0.0013393 | 0.18 | 74.32 |
| rs2760215 | T | C | -0.024985 | 2.44E-03 | 0.5034 | 0.487571 | 0.0038153 | 5.81E-11 | 0.0013203 | 0.065 | 21.44 |
| rs28530689 | A | C | 0.032213 | -0.003993 | 0.5117 | 0.524122 | 0.0038922 | 1.27E-16 | 0.0013216 | 0.0025 | 34.23 |
| rs2867112 | T | G | 0.035023 | -0.001777 | 0.8301 | 0.835297 | 0.0051437 | 9.84E-12 | 0.0018001 | 0.32 | 13.08 |
| rs2868194 | T | C | -0.026759 | -0.00054 | 0.4084 | 0.407593 | 0.0039217 | 8.90E-12 | 0.0013487 | 0.69 | 22.50 |
| rs2943539 | T | C | 0.04144 | 0.0003086 | 0.4754 | 0.478613 | 0.0037834 | 6.42E-28 | 0.0013194 | 0.82 | 59.85 |
| rs34861762 | T | C | 0.034282 | 0.0035078 | 0.4194 | 0.414017 | 0.0038297 | 3.50E-19 | 0.0013368 | 0.0087 | 39.03 |
| rs35198068 | T | C | 0.024734 | -1.84E-05 | 0.7063 | 0.708966 | 0.0042491 | 5.85E-09 | 0.0014533 | 0.99 | 14.06 |
| rs35396326 | C | G | 0.024742 | 0.0010726 | 0.704 | 0.720194 | 0.0044281 | 2.31E-08 | 0.0014919 | 0.47 | 13.01 |
| rs3925584 | T | C | 0.030389 | 0.0010662 | 0.5522 | 0.544461 | 0.0038157 | 1.66E-15 | 0.0013229 | 0.42 | 31.37 |
| rs455213 | T | C | -0.026532 | 0.0008257 | 0.5428 | 0.543711 | 0.0038574 | 6.05E-12 | 0.0013244 | 0.53 | 23.48 |
| rs4617927 | T | G | 0.035037 | -0.002546 | 0.446 | 0.431585 | 0.0039267 | 4.55E-19 | 0.0013413 | 0.058 | 39.35 |
| rs4646068 | T | C | 0.02366 | -0.001613 | 0.692 | 0.68536 | 0.0040972 | 7.71E-09 | 0.0014175 | 0.26 | 14.22 |
| rs4808762 | T | C | -0.024229 | 0.0030214 | 0.7199 | 0.710231 | 0.0042668 | 1.36E-08 | 0.0014562 | 0.038 | 13.00 |
| rs4897160 | A | G | 0.029735 | -0.00112 | 0.4828 | 0.476956 | 0.0038854 | 1.96E-14 | 0.001321 | 0.4 | 29.25 |
| rs4997081 | C | G | -0.030192 | -0.000582 | 0.1962 | 0.193202 | 0.0048328 | 4.18E-10 | 0.0016731 | 0.73 | 12.31 |
| rs57652769 | T | C | -0.036197 | -0.000279 | 0.3094 | 0.313728 | 0.0042129 | 8.56E-18 | 0.0014231 | 0.84 | 31.55 |
| rs6119510 | T | G | -0.023095 | -0.000398 | 0.5959 | 0.599016 | 0.0039005 | 3.20E-09 | 0.0013491 | 0.77 | 16.89 |
| rs62052820 | A | G | 0.041401 | 0.0039815 | 0.2124 | 0.229251 | 0.0047484 | 2.811E-18 | 0.0015709 | 0.011 | 25.44 |
| rs62294340 | A | G | -0.021858 | 0.0003098 | 0.364 | 0.383696 | 0.0040096 | 5.00E-08 | 0.0013557 | 0.82 | 13.76 |
| rs62310614 | A | G | -0.056289 | 0.000876 | 0.1104 | 0.108983 | 0.0062707 | 2.80E-19 | 0.002117 | 0.68 | 15.83 |
| rs62435145 | T | G | 0.041662 | -0.000417 | 0.6891 | 0.691513 | 0.0050793 | 2.36E-16 | 0.0014728 | 0.78 | 28.83 |
| rs626277 | A | C | 0.025914 | -0.002262 | 0.5938 | 0.602957 | 0.0038894 | 2.69E-11 | 0.0013483 | 0.093 | 21.42 |
| rs6283 | T | C | 0.043905 | 0.000979 | 0.6605 | 0.660733 | 0.0048971 | 3.09E-19 | 0.0014497 | 0.5 | 36.05 |
| rs6495044 | A | G | 0.024401 | 0.0001566 | 0.7086 | 0.695231 | 0.004281 | 1.20E-08 | 0.0014389 | 0.91 | 13.42 |
| rs7126110 | C | G | -0.074297 | -0.006096 | 0.8554 | 0.860031 | 0.0062508 | 1.40E-32 | 0.0019521 | 0.0018 | 34.95 |
| rs71456318 | A | C | 0.079027 | -0.000535 | 0.4842 | 0.451479 | 0.0038829 | 4.41E-92 | 0.0013261 | 0.69 | 207.05 |
| rs7267595 | A | C | 0.022524 | -3.70E-03 | 0.5097 | 0.526776 | 0.0038025 | 3.15E-09 | 0.0013274 | 0.0053 | 17.54 |
| rs72782806 | A | G | 0.025282 | -0.000689 | 0.2595 | 0.244275 | 0.0043848 | 8.12E-09 | 0.0015346 | 0.65 | 12.78 |
| rs7303595 | A | T | 0.025313 | 0.0032664 | 0.3357 | 0.323897 | 0.004106 | 7.05E-10 | 0.0014179 | 0.021 | 16.95 |
| rs7640441 | A | C | -0.027525 | -0.002349 | 0.2457 | 0.240032 | 0.0045325 | 1.26E-09 | 0.0015537 | 0.13 | 13.67 |
| rs7986094 | A | C | -0.023916 | -0.001542 | 0.3016 | 0.301258 | 0.0042434 | 1.74E-08 | 0.0014455 | 0.29 | 13.38 |
| rs8050136 | A | C | 0.02464 | -0.008249 | 0.4029 | 0.394212 | 0.0038882 | 2.34E-10 | 0.0013495 | 9.8E-10 | 19.32 |
| rs861536 | A | G | 0.0238 | 0.0034099 | 0.621 | 0.625509 | 0.0039764 | 2.16E-09 | 0.0013652 | 0.013 | 16.86 |
| rs9287911 | A | T | 0.038206 | 3.216E-05 | 0.2497 | 0.248597 | 0.0044632 | 1.126E-17 | 0.0015338 | 0.98 | 27.46 |
| rs9288447 | T | C | -0.022527 | -0.001199 | 0.5458 | 0.543031 | 0.0038068 | 3.269E-09 | 0.0013253 | 0.37 | 17.36 |
| rs9420446 | T | C | -0.038016 | 0.0012233 | 0.1371 | 0.150491 | 0.0055996 | 1.129E-11 | 0.0018478 | 0.51 | 10.91 |
| rs98270 | A | G | 0.021718 | -0.000657 | 0.362 | 0.363408 | 0.0039618 | 4.207E-08 | 0.0013782 | 0.63 | 13.88 |
| rs9895661 | T | C | 0.050208 | 0.0012005 | 0.8174 | 0.831329 | 0.0051 | 7.229E-23 | 0.0017648 | 0.5 | 28.93 |
| rs9925837 | A | G | -0.041628 | 0.0004457 | 0.8445 | 0.850237 | 0.0053322 | 5.854E-15 | 0.0018474 | 0.81 | 16.01 |

Two SNPs (rs1260326, rs35198068) that were removed from PhenoScanner step, One SNP (rs2581817) that were removed from harmonisation step, and one SNPs (rs8050136) that was removed from MR-PRESSO step.

**Table S8** Power of the models and F-statistics of the instrumental SNPs.

| **MR analysis Plink clumping using R2 < 0.1, p < 5E-8** | | | | | | | |
| --- | --- | --- | --- | --- | --- | --- | --- |
| **Exposure** | **Sample size** | **α** | **K** | **OR** | **R2xz** | **Power** | **F-statistic** |
| eGFR_cre | 1,004,040 | 0.050 | 3.48E-01 | 1.046 | 6.61E-03 | 0.42 | 6681.86 |
| eGFR_cys | 460,826 | 0.050 | 3.48E-01 | 0.997 | 1.00E-02 | 0.05 | 4655.81 |
| UACR | 288,649 | 0.050 | 3.48E-01 | 1.038 | 1.70E-03 | 0.07 | 492.54 |
| Urate | 547,361 | 0.050 | 3.48E-01 | 0.999 | 1.21E-02 | 0.05 | 6705.19 |

**Table S9** Effect estimates for the associations of the genetic variants with hearing loss and eGFR_cre.

| **SNP** | **effect_allele** | **other_allele** | **beta.exposure** | **beta.outcome** | **eaf.exposure** | **eaf.outcome** | **se.outcome** | **pval.outcome** | **se.exposure** | **pval.exposure** | **F_statisic** |
| --- | --- | --- | --- | --- | --- | --- | --- | --- | --- | --- | --- |
| rs10477835 | A | T | -0.0054 | 0 | 0.3818 | 0.3848 | 3.00E-04 | 9.39E-01 | 0.001 | 1.431E-08 | 13.765844 |
| rs10901863 | T | C | 0.0106 | 0.0005 | 0.2683 | 0.2741 | 3.00E-04 | 1.45E-01 | 0.0011 | 9.295E-23 | 36.464466 |
| rs10948071 | T | C | 0.0091 | -0.0002 | 0.6098 | 0.6046 | 3.00E-04 | 4.44E-01 | 0.001 | 1.485E-21 | 39.414167 |
| rs1097215 | A | G | -0.0053 | -0.0021 | 0.4752 | 0.4787 | 3.00E-04 | 9.74E-12 | 0.0009 | 1.113E-08 | 17.297905 |
| rs11075990 | A | G | 0.0054 | -0.0001 | 0.6056 | 0.6029 | 3.00E-04 | 7.56E-01 | 0.001 | 2.637E-08 | 13.930316 |
| rs11238325 | T | C | 0.007 | 0.0001 | 0.7315 | 0.7223 | 3.00E-04 | 7.54E-01 | 0.001 | 1.969E-11 | 19.249285 |
| rs1126809 | A | G | 0.0093 | 0.0003 | 0.3005 | 0.2932 | 3.00E-04 | 4.39E-01 | 0.001 | 2.152E-20 | 36.365343 |
| rs11881070 | T | C | -0.006 | 0.0003 | 0.2882 | 0.2895 | 3.00E-04 | 4.58E-01 | 0.001 | 5.72E-09 | 14.770888 |
| rs12441297 | T | G | 0.0079 | 0.0002 | 0.8043 | 0.8068 | 3.00E-04 | 5.24E-01 | 0.0012 | 1.544E-11 | 13.644285 |
| rs13147559 | C | G | -0.0098 | 0.001 | 0.8662 | 0.863 | 4.00E-04 | 2.32E-02 | 0.0014 | 6.69E-13 | 11.358385 |
| rs13171669 | A | G | -0.0063 | -0.0005 | 0.5682 | 0.5788 | 3.00E-04 | 8.02E-02 | 0.0009 | 1.609E-11 | 24.046296 |
| rs13268718 | T | G | -0.0054 | -0.0002 | 0.5072 | 0.5071 | 3.00E-04 | 5.93E-01 | 0.0009 | 7.467E-09 | 17.997417 |
| rs13337678 | T | C | -0.0052 | 0.0007 | 0.5711 | 0.5671 | 3.00E-04 | 2.62E-02 | 0.0009 | 3.719E-08 | 16.354782 |
| rs1566128 | A | G | 0.0073 | 0 | 0.4126 | 0.4127 | 3.00E-04 | 9.79E-01 | 0.0009 | 1.422E-14 | 31.893759 |
| rs17671352 | T | C | 0.0061 | 0 | 0.379 | 0.3755 | 3.00E-04 | 9.15E-01 | 0.001 | 1.524E-10 | 17.516502 |
| rs2076371 | A | G | 0.0058 | -0.0001 | 0.2754 | 0.2736 | 3.00E-04 | 7.07E-01 | 0.001 | 2.935E-08 | 13.426662 |
| rs2393729 | T | C | -0.0059 | 0.0013 | 0.4218 | 0.4208 | 3.00E-04 | 2.59E-05 | 0.0009 | 3.067E-10 | 20.963633 |
| rs2703636 | T | C | -0.0072 | -0.0004 | 0.7233 | 0.7265 | 3.00E-04 | 2.18E-01 | 0.001 | 5.089E-12 | 20.75177 |
| rs2877561 | A | C | 0.0062 | 0.0023 | 0.2724 | 0.2761 | 3.00E-04 | 4.67E-12 | 0.0011 | 3.519E-09 | 12.593492 |
| rs323693 | T | C | -0.0102 | -0.0004 | 0.882 | 0.8812 | 4.00E-04 | 3.69E-01 | 0.0014 | 1.914E-12 | 11.049447 |
| rs36062310 | A | G | 0.0274 | 0 | 0.0427 | 0.0423 | 7.00E-04 | 9.67E-01 | 0.0023 | 4.25E-32 | 11.60294 |
| rs4413512 | A | G | -0.0098 | -0.0001 | 0.5289 | 0.5345 | 3.00E-04 | 7.33E-01 | 0.0009 | 1.283E-25 | 59.099367 |
| rs4483583 | A | C | 0.0089 | 0.0007 | 0.8034 | 8.03E-01 | 3.00E-04 | 5.63E-02 | 0.0012 | 1.964E-14 | 17.377589 |
| rs4660885 | A | G | -0.0065 | 0.0012 | 0.4344 | 0.4431 | 3.00E-04 | 4.87E-05 | 0.0009 | 3.737E-12 | 25.633735 |
| rs4732339 | A | G | 0.0058 | 0.0001 | 0.5864 | 0.5872 | 3.00E-04 | 6.60E-01 | 0.0009 | 6.096E-10 | 20.14684 |
| rs521539 | A | G | 0.0079 | 0.0017 | 0.2145 | 0.2114 | 3.00E-04 | 9.99E-06 | 0.0012 | 1.768E-11 | 14.60552 |
| rs566673 | T | G | -0.0051 | -0.0005 | 0.5339 | 0.5373 | 3.00E-04 | 1.26E-01 | 0.0009 | 3.407E-08 | 15.982643 |
| rs5756795 | T | C | -0.0079 | 0.0003 | 0.5419 | 0.5505 | 3.00E-04 | 3.32E-01 | 0.0009 | 3.645E-17 | 38.259694 |
| rs62015206 | T | C | 0.0053 | -0.0002 | 0.5903 | 0.5915 | 3.00E-04 | 5.53E-01 | 0.001 | 3.165E-08 | 13.587532 |
| rs6455991 | T | C | -0.0061 | -0.0006 | 0.4852 | 0.4832 | 3.00E-04 | 3.44E-02 | 0.001 | 1.71E-10 | 18.589931 |
| rs6545432 | A | G | 0.0068 | 0.0005 | 0.5091 | 0.5007 | 3.00E-04 | 6.86E-02 | 0.0009 | 2.356E-13 | 28.536779 |
| rs67307131 | T | C | -0.0079 | 0.0007 | 0.654 | 0.6566 | 3.00E-04 | 3.48E-02 | 0.001 | 4.619E-15 | 28.24773 |
| rs72622585 | T | C | 0.0091 | 0.0003 | 0.8252 | 0.8222 | 4.00E-04 | 4.21E-01 | 0.0013 | 3.405E-13 | 14.136691 |
| rs72930998 | T | C | -0.007 | 0.0002 | 0.7862 | 0.7872 | 3.00E-04 | 5.03E-01 | 0.0011 | 5.243E-10 | 13.614497 |
| rs7313797 | T | C | -0.0064 | 0.0002 | 0.5604 | 0.5546 | 3.00E-04 | 5.49E-01 | 0.0009 | 7.382E-12 | 24.917271 |
| rs741475 | T | C | -0.0059 | 0.0008 | 0.5771 | 0.5733 | 3.00E-04 | 1.12E-02 | 0.0009 | 4.018E-10 | 20.978319 |
| rs7525101 | T | C | 0.0061 | 0.0002 | 0.4424 | 0.4446 | 3.00E-04 | 4.30E-01 | 0.0009 | 8.643E-11 | 22.666182 |
| rs78417468 | A | G | -0.0068 | 0 | 0.2242 | 2.23E-01 | 3.00E-04 | 9.80E-01 | 0.0011 | 8.839E-10 | 13.29436 |
| rs7926527 | T | C | 0.0057 | 0.0003 | 0.6424 | 0.6401 | 3.00E-04 | 4.11E-01 | 0.001 | 3.346E-09 | 14.928122 |
| rs920701 | T | C | -0.0064 | -0.0006 | 0.6357 | 0.6388 | 3.00E-04 | 4.05E-02 | 0.001 | 5.06E-11 | 18.972771 |
| rs9493627 | A | G | 0.0085 | 0.0029 | 0.3191 | 0.3198 | 3.00E-04 | 9.67E-19 | 0.001 | 9.563E-18 | 31.399951 |
| rs9517282 | A | C | -0.0052 | -0.0004 | 0.548 | 0.5497 | 0.0003 | 0.2195 | 0.0009 | 3.541E-08 | 16.538491 |

1 SNP (rs521539) that were removed from harmonization step, and 7 SNPs (rs1097215, rs13147559, rs2393729, rs2877561, rs4660885, rs741475, rs9493627) that were removed from MR-PRESSO step.

**Table S10** Effect estimates for the associations of the genetic variants with hearing loss and eGFR_cys.

| **SNP** | **effect_allele** | **other_allele** | **beta.exposure** | **beta.outcome** | **eaf.exposure** | **eaf.outcome** | **se.exposure** | **pval.exposure** | **se.outcome** | **pval.outcome** | **F_statisic** |
| --- | --- | --- | --- | --- | --- | --- | --- | --- | --- | --- | --- |
| rs10477835 | A | T | -0.0054 | 0.0015 | 0.3818 | 0.3786 | 0.001 | 1.431E-08 | 5.00E-04 | 2.78E-03 | 13.765844 |
| rs10901863 | T | C | 0.0106 | 0.0006 | 0.2683 | 0.2671 | 0.0011 | 9.295E-23 | 5.00E-04 | 3.31E-01 | 36.464466 |
| rs10948071 | T | C | 0.0091 | -0.0051 | 0.6098 | 0.6032 | 0.001 | 1.485E-21 | 5.00E-04 | 7.70E-24 | 39.414167 |
| rs1097215 | A | G | -0.0053 | -0.0024 | 0.4752 | 0.4886 | 0.0009 | 1.113E-08 | 5.00E-04 | 1.29E-06 | 17.297905 |
| rs11075990 | A | G | 0.0054 | 0.004 | 0.6056 | 0.6073 | 0.001 | 2.637E-08 | 5.00E-04 | 1.91E-15 | 13.930316 |
| rs11238325 | T | C | 0.007 | -0.0003 | 0.7315 | 0.7269 | 0.001 | 1.969E-11 | 5.00E-04 | 6.15E-01 | 19.249285 |
| rs1126809 | A | G | 0.0093 | 0 | 0.3005 | 0.2973 | 0.001 | 2.152E-20 | 5.00E-04 | 9.97E-01 | 36.365343 |
| rs11881070 | T | C | -0.006 | 0.0003 | 0.2882 | 0.2901 | 0.001 | 5.72E-09 | 5.00E-04 | 6.49E-01 | 14.770888 |
| rs12441297 | T | G | 0.0079 | 0.0004 | 0.8043 | 0.8029 | 0.0012 | 1.544E-11 | 6.00E-04 | 4.95E-01 | 13.644285 |
| rs13147559 | C | G | -0.0098 | 0.0002 | 0.8662 | 0.8673 | 0.0014 | 6.69E-13 | 7.00E-04 | 7.50E-01 | 11.358385 |
| rs13171669 | A | G | -0.0063 | 0.0006 | 0.5682 | 0.5731 | 0.0009 | 1.609E-11 | 5.00E-04 | 2.57E-01 | 24.046296 |
| rs13268718 | T | G | -0.0054 | 0.0001 | 0.5072 | 0.5031 | 0.0009 | 7.467E-09 | 5.00E-04 | 8.80E-01 | 17.997417 |
| rs13337678 | T | C | -0.0052 | 0.0002 | 0.5711 | 0.575 | 0.0009 | 3.719E-08 | 5.00E-04 | 6.32E-01 | 16.354782 |
| rs1566128 | A | G | 0.0073 | -0.0007 | 0.4126 | 0.4186 | 0.0009 | 1.422E-14 | 5.00E-04 | 1.68E-01 | 31.893759 |
| rs17671352 | T | C | 0.0061 | 0.0002 | 0.379 | 0.3841 | 0.001 | 1.524E-10 | 5.00E-04 | 6.56E-01 | 17.516502 |
| rs2076371 | A | G | 0.0058 | 0.0003 | 0.2754 | 0.2731 | 0.001 | 2.935E-08 | 5.00E-04 | 5.56E-01 | 13.426662 |
| rs2393729 | T | C | -0.0059 | 0.0009 | 0.4218 | 0.4171 | 0.0009 | 3.067E-10 | 5.00E-04 | 7.02E-02 | 20.963633 |
| rs2703636 | T | C | -0.0072 | -0.0003 | 0.7233 | 0.7274 | 0.001 | 5.089E-12 | 5.00E-04 | 5.50E-01 | 20.75177 |
| rs2877561 | A | C | 0.0062 | 0.0021 | 0.2724 | 0.2701 | 0.0011 | 3.519E-09 | 5.00E-04 | 2.55E-04 | 12.593492 |
| rs323693 | T | C | -0.0102 | -0.0004 | 0.882 | 0.8803 | 0.0014 | 1.914E-12 | 7.00E-04 | 5.95E-01 | 11.049447 |
| rs36062310 | A | G | 0.0274 | 0.0001 | 0.0427 | 0.0426 | 0.0023 | 4.25E-32 | 1.10E-03 | 9.59E-01 | 11.60294 |
| rs4413512 | A | G | -0.0098 | -0.0001 | 0.5289 | 0.5332 | 0.0009 | 1.283E-25 | 5.00E-04 | 9.09E-01 | 59.099367 |
| rs4483583 | A | C | 0.0089 | 0.0012 | 0.8034 | 8.07E-01 | 0.0012 | 1.964E-14 | 6.00E-04 | 6.34E-02 | 17.377589 |
| rs4660885 | A | G | -0.0065 | 0.0026 | 0.4344 | 0.4414 | 0.0009 | 3.737E-12 | 5.00E-04 | 3.39E-07 | 25.633735 |
| rs4732339 | A | G | 0.0058 | 0.0004 | 0.5864 | 0.583 | 0.0009 | 6.096E-10 | 5.00E-04 | 4.56E-01 | 20.14684 |
| rs566673 | T | G | -0.0051 | 0.0014 | 0.5339 | 0.5398 | 0.0009 | 3.407E-08 | 5.00E-04 | 5.83E-03 | 15.982643 |
| rs5756795 | T | C | -0.0079 | 0.0004 | 0.5419 | 0.5451 | 0.0009 | 3.645E-17 | 5.00E-04 | 4.83E-01 | 38.259694 |
| rs62015206 | T | C | 0.0053 | -0.0004 | 0.5903 | 0.5932 | 0.001 | 3.165E-08 | 5.00E-04 | 4.09E-01 | 13.587532 |
| rs6455991 | T | C | -0.0061 | -0.0006 | 0.4852 | 0.4843 | 0.001 | 1.71E-10 | 5.00E-04 | 2.60E-01 | 18.589931 |
| rs6545432 | A | G | 0.0068 | 0.0006 | 0.5091 | 0.5132 | 0.0009 | 2.356E-13 | 5.00E-04 | 2.56E-01 | 28.536779 |
| rs67307131 | T | C | -0.0079 | 0.0004 | 0.654 | 0.6518 | 0.001 | 4.619E-15 | 5.00E-04 | 4.59E-01 | 28.24773 |
| rs72622585 | T | C | 0.0091 | 0.0009 | 0.8252 | 0.8192 | 0.0013 | 3.405E-13 | 6.00E-04 | 1.57E-01 | 14.136691 |
| rs72930998 | T | C | -0.007 | 0.001 | 0.7862 | 0.7863 | 0.0011 | 5.243E-10 | 6.00E-04 | 1.05E-01 | 13.614497 |
| rs7313797 | T | C | -0.0064 | 0.0012 | 0.5604 | 0.557 | 0.0009 | 7.382E-12 | 5.00E-04 | 1.37E-02 | 24.917271 |
| rs741475 | T | C | -0.0059 | 0.0015 | 0.5771 | 0.5751 | 0.0009 | 4.018E-10 | 5.00E-04 | 2.21E-03 | 20.978319 |
| rs7525101 | T | C | 0.0061 | 0.0003 | 0.4424 | 0.4389 | 0.0009 | 8.643E-11 | 5.00E-04 | 5.76E-01 | 22.666182 |
| rs78417468 | A | G | -0.0068 | 0.0001 | 0.2242 | 0.2236 | 0.0011 | 8.839E-10 | 6.00E-04 | 8.97E-01 | 13.29436 |
| rs7926527 | T | C | 0.0057 | -0.0002 | 0.6424 | 6.39E-01 | 0.001 | 3.346E-09 | 5.00E-04 | 7.29E-01 | 14.928122 |
| rs920701 | T | C | -0.0064 | -0.001 | 0.6357 | 0.6333 | 0.001 | 5.06E-11 | 5.00E-04 | 5.39E-02 | 18.972771 |
| rs9493627 | A | G | 0.0085 | -0.0005 | 0.3191 | 0.3227 | 0.001 | 9.563E-18 | 5.00E-04 | 3.12E-01 | 31.399951 |
| rs9517282 | A | C | -0.0052 | -0.0012 | 0.548 | 0.5441 | 0.0009 | 3.541E-08 | 5.00E-04 | 1.86E-02 | 16.538491 |

5 SNPs (rs10948071, rs1097215, rs11075990, rs2877561, rs4660885) that were removed from MR-PRESSO step.

**Table S11** Effect estimates for the associations of the genetic variants with hearing loss and UACR.

| **SNP** | **effect_allele** | **other_allele** | **beta.exposure** | **beta.outcome** | **eaf.exposure** | **eaf.outcome** | **se.exposure** | **pval.exposure** | **se.outcome** | **pval.outcome** | **F_statisic** |
| --- | --- | --- | --- | --- | --- | --- | --- | --- | --- | --- | --- |
| rs10477835 | A | T | -0.0054 | 0.0038254 | 0.3818 | 0.3826 | 1.00E-03 | 1.43E-08 | 0.0020462 | 0.0615513 | 13.765844 |
| rs10901863 | T | C | 0.0106 | -0.002823 | 0.2683 | 0.2698 | 1.10E-03 | 9.30E-23 | 0.0023723 | 0.2340525 | 36.464466 |
| rs10948071 | T | C | 0.0091 | -0.002682 | 0.6098 | 0.6049 | 1.00E-03 | 1.49E-21 | 0.0020248 | 0.1853121 | 39.414167 |
| rs1097215 | A | G | -0.0053 | -0.0091369 | 0.4752 | 0.4793 | 9.00E-04 | 1.11E-08 | 0.0020232 | 6.3E-06 | 17.297905 |
| rs11075990 | A | G | 0.0054 | 0.001572 | 0.6056 | 0.6028 | 1.00E-03 | 2.64E-08 | 0.0020267 | 0.4379585 | 13.930316 |
| rs11238325 | T | C | 0.007 | -0.0015186 | 0.7315 | 0.7328 | 1.00E-03 | 1.97E-11 | 0.0022451 | 0.4987827 | 19.249285 |
| rs1126809 | A | G | 0.0093 | 0.0026034 | 0.3005 | 0.2968 | 1.00E-03 | 2.15E-20 | 0.0022126 | 0.2393453 | 36.365343 |
| rs11881070 | T | C | -0.006 | 0.0026364 | 0.2882 | 0.2883 | 1.00E-03 | 5.72E-09 | 0.0023009 | 0.2518727 | 14.770888 |
| rs12441297 | T | G | 0.0079 | -0.0020916 | 0.8043 | 0.803 | 1.20E-03 | 1.54E-11 | 0.0025432 | 0.4108331 | 13.644285 |
| rs13147559 | C | G | -0.0098 | 0.0010477 | 0.8662 | 0.866 | 1.40E-03 | 6.69E-13 | 0.0029632 | 0.7236608 | 11.358385 |
| rs13171669 | A | G | -0.0063 | -0.0003214 | 0.5682 | 0.5733 | 9.00E-04 | 1.61E-11 | 0.0020439 | 0.8750491 | 24.046296 |
| rs13268718 | T | G | -0.0054 | 0.0061266 | 0.5072 | 0.5043 | 9.00E-04 | 7.47E-09 | 0.0020032 | 0.0022252 | 17.997417 |
| rs13337678 | T | C | -0.0052 | -0.0012189 | 0.5711 | 0.5719 | 9.00E-04 | 3.72E-08 | 0.0020312 | 0.5484472 | 16.354782 |
| rs1566128 | A | G | 0.0073 | 0.0016761 | 0.4126 | 0.414 | 9.00E-04 | 1.42E-14 | 0.002015 | 0.4055154 | 31.893759 |
| rs17671352 | T | C | 0.0061 | -0.0062002 | 0.379 | 0.376 | 1.00E-03 | 1.52E-10 | 0.0020516 | 0.00251 | 17.516502 |
| rs2076371 | A | G | 0.0058 | -0.0026911 | 0.2754 | 0.2764 | 1.00E-03 | 2.94E-08 | 0.0022788 | 0.2376311 | 13.426662 |
| rs2393729 | T | C | -0.0059 | 0.000429 | 0.4218 | 0.4196 | 9.00E-04 | 3.07E-10 | 0.0020129 | 0.8312292 | 20.963633 |
| rs2703636 | T | C | -0.0072 | -0.0031715 | 0.7233 | 0.7247 | 1.00E-03 | 5.09E-12 | 0.0022657 | 0.1615768 | 20.75177 |
| rs2877561 | A | C | 0.0062 | 0.0079345 | 0.2724 | 0.2743 | 1.10E-03 | 3.52E-09 | 0.0022361 | 0.0003876 | 12.593492 |
| rs323693 | T | C | -0.0102 | -0.000592 | 0.882 | 0.8819 | 1.40E-03 | 1.91E-12 | 0.0030823 | 0.8476918 | 11.049447 |
| rs36062310 | A | G | 0.0274 | 0.0027341 | 0.0427 | 0.0429 | 2.30E-03 | 4.25E-32 | 0.0051694 | 0.5968742 | 11.60294 |
| rs4413512 | A | G | -0.0098 | 0.0019388 | 0.5289 | 0.5328 | 9.00E-04 | 1.28E-25 | 0.0020197 | 0.3370831 | 59.099367 |
| rs4483583 | A | C | 0.0089 | -1.49E-03 | 0.8034 | 0.8039 | 1.20E-03 | 1.96E-14 | 0.0025362 | 0.5567138 | 17.377589 |
| rs4660885 | A | G | -0.0065 | 0.0075174 | 0.4344 | 0.4392 | 9.00E-04 | 3.74E-12 | 0.0020259 | 0.0002067 | 25.633735 |
| rs4732339 | A | G | 0.0058 | -0.0006937 | 0.5864 | 0.5868 | 9.00E-04 | 6.10E-10 | 0.0020151 | 0.7306576 | 20.14684 |
| rs521539 | A | G | 0.0079 | 0.0019495 | 0.2145 | 0.2096 | 1.20E-03 | 1.77E-11 | 0.0024913 | 0.4339072 | 14.60552 |
| rs566673 | T | G | -0.0051 | -0.0002434 | 0.5339 | 0.536 | 9.00E-04 | 3.41E-08 | 0.0020048 | 0.9033674 | 15.982643 |
| rs5756795 | T | C | -0.0079 | 0.0045522 | 0.5419 | 0.5469 | 9.00E-04 | 3.65E-17 | 0.0020167 | 0.0239923 | 38.259694 |
| rs62015206 | T | C | 0.0053 | 0.0002095 | 0.5903 | 0.5905 | 1.00E-03 | 3.17E-08 | 0.002069 | 0.9193467 | 13.587532 |
| rs6455991 | T | C | -0.0061 | -0.0012492 | 0.4852 | 0.4859 | 1.00E-03 | 1.71E-10 | 0.0019895 | 0.5300718 | 18.589931 |
| rs6545432 | A | G | 0.0068 | -0.0023952 | 0.5091 | 0.5068 | 9.00E-04 | 2.36E-13 | 0.001984 | 0.2273328 | 28.536779 |
| rs67307131 | T | C | -0.0079 | -0.0012461 | 0.654 | 0.6551 | 1.00E-03 | 4.62E-15 | 0.0021372 | 0.5598579 | 28.24773 |
| rs72622585 | T | C | 0.0091 | 0.009053 | 0.8252 | 0.8231 | 1.30E-03 | 3.41E-13 | 0.0026384 | 0.0006008 | 14.136691 |
| rs72930998 | T | C | -0.007 | 0.0042665 | 0.7862 | 0.7832 | 1.10E-03 | 5.24E-10 | 0.0024107 | 0.0767575 | 13.614497 |
| rs7313797 | T | C | -0.0064 | 0.0017256 | 0.5604 | 0.5566 | 9.00E-04 | 7.38E-12 | 0.0019993 | 0.3880813 | 24.917271 |
| rs741475 | T | C | -0.0059 | -0.0013116 | 0.5771 | 0.5743 | 9.00E-04 | 4.02E-10 | 0.0020147 | 0.5150368 | 20.978319 |
| rs7525101 | T | C | 0.0061 | -0.0013269 | 0.4424 | 0.4408 | 9.00E-04 | 8.64E-11 | 0.0020303 | 0.5134026 | 22.666182 |
| rs78417468 | A | G | -0.0068 | -1.90E-03 | 0.2242 | 0.225 | 1.10E-03 | 8.84E-10 | 0.0023765 | 0.4231503 | 13.29436 |
| rs7926527 | T | C | 0.0057 | -0.0014782 | 0.6424 | 0.6411 | 1.00E-03 | 3.35E-09 | 0.0020952 | 0.4804883 | 14.928122 |
| rs920701 | T | C | -0.0064 | -0.006453 | 0.6357 | 0.6386 | 1.00E-03 | 5.06E-11 | 0.0020654 | 0.0017821 | 18.972771 |
| rs9493627 | A | G | 0.0085 | 0.0027294 | 0.3191 | 0.3207 | 1.00E-03 | 9.56E-18 | 0.0021241 | 0.1988036 | 31.399951 |
| rs9517282 | A | C | -0.0052 | 0.000847 | 0.548 | 0.5477 | 0.0009 | 3.541E-08 | 0.0020204 | 0.6750525 | 16.538491 |

5 SNPs (rs1097215, rs2877561, rs4660885, rs72622585, rs920701) that were removed from MR-PRESSO step.

**Table S12** Effect estimates for the associations of the genetic variants with hearing loss and urate.

| **SNP** | **effect_allele** | **other_allele** | **beta.exposure** | **beta.outcome** | **eaf.exposure** | **eaf.outcome** | **se.exposure** | **pval.exposure** | **se.outcome** | **pval.outcome** | **F_statisic** |
| --- | --- | --- | --- | --- | --- | --- | --- | --- | --- | --- | --- |
| rs10477835 | A | T | -0.0054 | -0.003703 | 0.3818 | 0.3927 | 0.001 | 1.431E-08 | 3.90E-03 | 3.43E-01 | 13.765844 |
| rs10901863 | T | C | 0.0106 | 0.003072 | 0.2683 | 0.2757 | 0.0011 | 9.295E-23 | 4.95E-03 | 5.35E-01 | 36.464466 |
| rs10948071 | T | C | 0.0091 | -0.028939 | 0.6098 | 0.5951 | 0.001 | 1.485E-21 | 3.84E-03 | 4.93E-14 | 39.414167 |
| rs1097215 | A | G | -0.0053 | 0.006419 | 0.4752 | 0.4903 | 0.0009 | 1.113E-08 | 3.91E-03 | 1.01E-01 | 17.297905 |
| rs11075990 | A | G | 0.0054 | -0.022795 | 0.6056 | 0.5963 | 0.001 | 2.637E-08 | 3.85E-03 | 3.07E-09 | 13.930316 |
| rs11238325 | T | C | 0.007 | -0.003974 | 0.7315 | 0.7236 | 0.001 | 1.969E-11 | 4.29E-03 | 3.54E-01 | 19.249285 |
| rs1126809 | A | G | 0.0093 | 0.002731 | 0.3005 | 0.2695 | 0.001 | 2.152E-20 | 4.40E-03 | 5.34E-01 | 36.365343 |
| rs11881070 | T | C | -0.006 | 0.011988 | 0.2882 | 0.2865 | 0.001 | 5.72E-09 | 4.77E-03 | 1.20E-02 | 14.770888 |
| rs12441297 | T | G | 0.0079 | -0.000375 | 0.8043 | 0.8003 | 0.0012 | 1.544E-11 | 4.85E-03 | 9.38E-01 | 13.644285 |
| rs13147559 | C | G | -0.0098 | -0.00483 | 0.8662 | 0.8665 | 0.0014 | 6.69E-13 | 6.57E-03 | 4.63E-01 | 11.358385 |
| rs13171669 | A | G | -0.0063 | -0.00775 | 0.5682 | 0.5945 | 0.0009 | 1.609E-11 | 3.99E-03 | 5.18E-02 | 24.046296 |
| rs13268718 | T | G | -0.0054 | 0.000793 | 0.5072 | 0.5002 | 0.0009 | 7.467E-09 | 3.91E-03 | 8.39E-01 | 17.997417 |
| rs13337678 | T | C | -0.0052 | -0.008608 | 0.5711 | 0.5641 | 0.0009 | 3.719E-08 | 3.92E-03 | 2.81E-02 | 16.354782 |
| rs1566128 | A | G | 0.0073 | -0.001974 | 0.4126 | 0.4116 | 0.0009 | 1.422E-14 | 3.89E-03 | 6.12E-01 | 31.893759 |
| rs17671352 | T | C | 0.0061 | -0.004026 | 0.379 | 0.3617 | 0.001 | 1.524E-10 | 3.99E-03 | 3.13E-01 | 17.516502 |
| rs2076371 | A | G | 0.0058 | 0.000863 | 0.2754 | 0.2728 | 0.001 | 2.935E-08 | 4.51E-03 | 8.48E-01 | 13.426662 |
| rs2393729 | T | C | -0.0059 | -0.002081 | 0.4218 | 0.4118 | 0.0009 | 3.067E-10 | 3.84E-03 | 5.88E-01 | 20.963633 |
| rs2703636 | T | C | -0.0072 | 0.004786 | 0.7233 | 0.7296 | 0.001 | 5.089E-12 | 4.38E-03 | 2.75E-01 | 20.75177 |
| rs2877561 | A | C | 0.0062 | -0.009371 | 0.2724 | 0.2846 | 0.0011 | 3.519E-09 | 4.23E-03 | 2.67E-02 | 12.593492 |
| rs323693 | T | C | -0.0102 | 0.002522 | 0.882 | 0.877 | 0.0014 | 1.914E-12 | 5.82E-03 | 6.65E-01 | 11.049447 |
| rs36062310 | A | G | 0.0274 | -0.027474 | 0.0427 | 0.0409 | 0.0023 | 4.25E-32 | 1.18E-02 | 2.02E-02 | 11.60294 |
| rs4413512 | A | G | -0.0098 | 0.00392 | 0.5289 | 0.5471 | 0.0009 | 1.283E-25 | 3.87E-03 | 3.11E-01 | 59.099367 |
| rs4483583 | A | C | 0.0089 | 0.004014 | 0.8034 | 7.99E-01 | 0.0012 | 1.964E-14 | 4.78E-03 | 4.01E-01 | 17.377589 |
| rs4660885 | A | G | -0.0065 | -0.005827 | 0.4344 | 0.4514 | 0.0009 | 3.737E-12 | 3.90E-03 | 1.35E-01 | 25.633735 |
| rs4732339 | A | G | 0.0058 | 0.008448 | 0.5864 | 0.5917 | 0.0009 | 6.096E-10 | 3.86E-03 | 2.87E-02 | 20.14684 |
| rs521539 | A | G | 0.0079 | -0.004709 | 0.2145 | 0.2004 | 0.0012 | 1.768E-11 | 5.26E-03 | 3.71E-01 | 14.60552 |
| rs566673 | T | G | -0.0051 | -0.000331 | 0.5339 | 0.5368 | 0.0009 | 3.407E-08 | 3.91E-03 | 9.33E-01 | 15.982643 |
| rs5756795 | T | C | -0.0079 | -0.004578 | 0.5419 | 0.565 | 0.0009 | 3.645E-17 | 3.89E-03 | 2.40E-01 | 38.259694 |
| rs62015206 | T | C | 0.0053 | -0.004059 | 0.5903 | 0.5877 | 0.001 | 3.165E-08 | 4.03E-03 | 3.14E-01 | 13.587532 |
| rs6455991 | T | C | -0.0061 | -0.000012 | 0.4852 | 0.4806 | 0.001 | 1.71E-10 | 3.83E-03 | 9.97E-01 | 18.589931 |
| rs6545432 | A | G | 0.0068 | -0.007003 | 0.5091 | 0.4956 | 0.0009 | 2.356E-13 | 3.85E-03 | 6.89E-02 | 28.536779 |
| rs67307131 | T | C | -0.0079 | -0.007323 | 0.654 | 0.658 | 0.001 | 4.619E-15 | 4.14E-03 | 7.72E-02 | 28.24773 |
| rs72622585 | T | C | 0.0091 | -0.008478 | 0.8252 | 0.8187 | 0.0013 | 3.405E-13 | 4.98E-03 | 8.86E-02 | 14.136691 |
| rs72930998 | T | C | -0.007 | 0.003456 | 0.7862 | 0.78 | 0.0011 | 5.243E-10 | 4.62E-03 | 4.54E-01 | 13.614497 |
| rs7313797 | T | C | -0.0064 | -0.004678 | 0.5604 | 0.548 | 0.0009 | 7.382E-12 | 3.83E-03 | 2.22E-01 | 24.917271 |
| rs741475 | T | C | -0.0059 | 0.005791 | 0.5771 | 0.5653 | 0.0009 | 4.018E-10 | 3.92E-03 | 1.39E-01 | 20.978319 |
| rs7525101 | T | C | 0.0061 | -0.002851 | 0.4424 | 0.4408 | 0.0009 | 8.643E-11 | 3.90E-03 | 4.65E-01 | 22.666182 |
| rs78417468 | A | G | -0.0068 | -0.004608 | 0.2242 | 2.24E-01 | 0.0011 | 8.839E-10 | 4.55E-03 | 3.11E-01 | 13.29436 |
| rs7926527 | T | C | 0.0057 | -0.00386 | 0.6424 | 0.6448 | 0.001 | 3.346E-09 | 4.21E-03 | 3.59E-01 | 14.928122 |
| rs920701 | T | C | -0.0064 | 0.001515 | 0.6357 | 0.6422 | 0.001 | 5.06E-11 | 3.96E-03 | 7.02E-01 | 18.972771 |
| rs9493627 | A | G | 0.0085 | -0.000654 | 0.3191 | 0.3254 | 0.001 | 9.563E-18 | 4.08E-03 | 8.73E-01 | 31.399951 |
| rs9517282 | A | C | -0.0052 | 0.010449 | 0.548 | 0.5421 | 0.0009 | 3.541E-08 | 0.003926 | 0.0077796 | 16.538491 |

2 SNPs (rs10948071, rs11075990) that were removed from MR-PRESSO step.

**Supplementary Figures**

**Figure S1**. Forest plots for the MR leave-one-out analysis of the significant IVW estimates.

**Figure S2.** Funnel plot of the MR analysis for causal association of renal function with hearing loss.

**Figure S1. Forest plots for the MR leave-one-out analysis of the significant IVW estimates.**


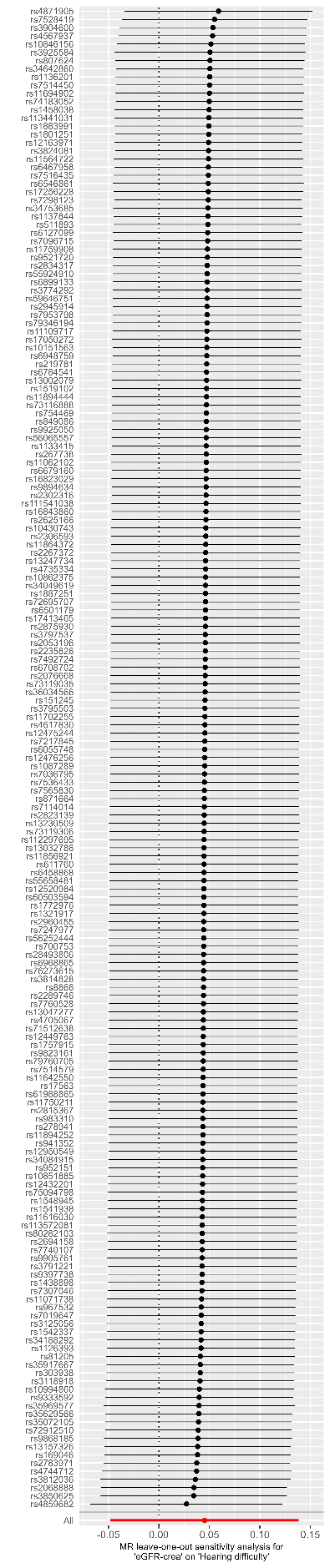

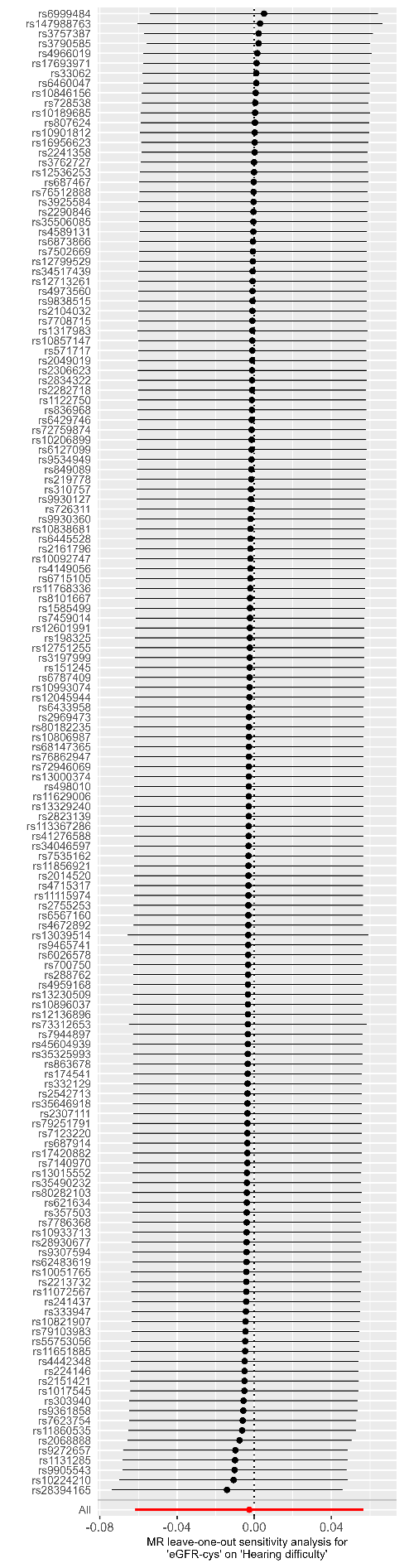


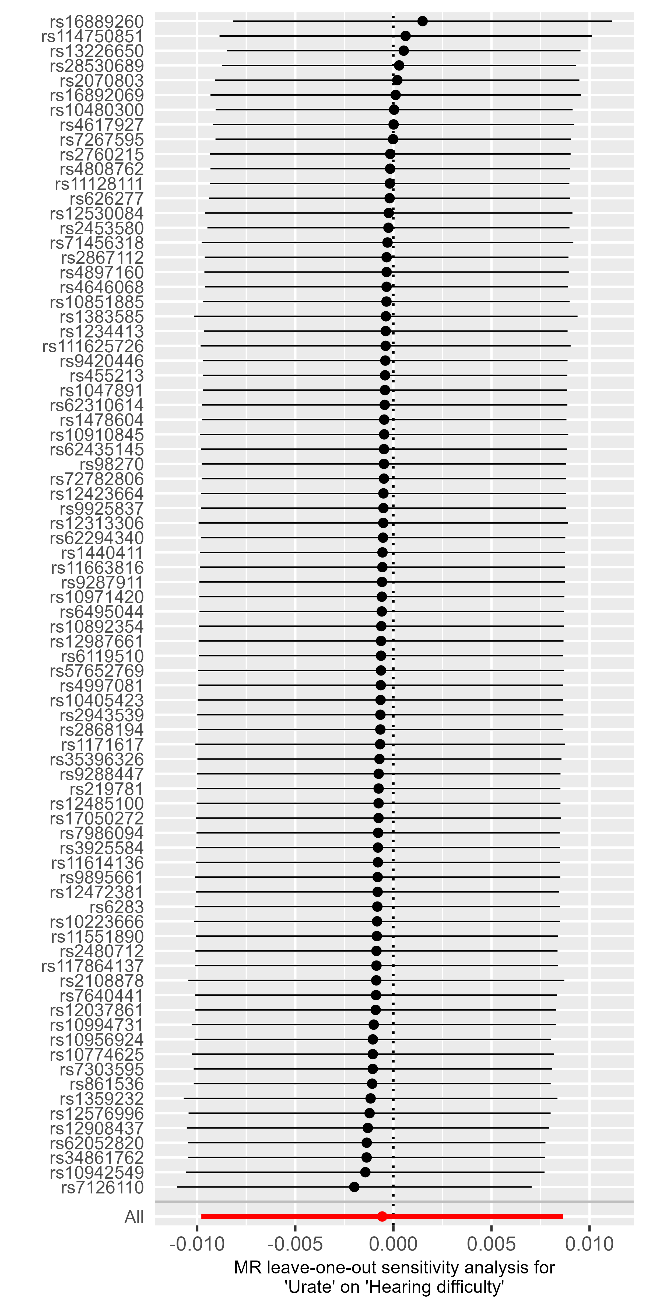

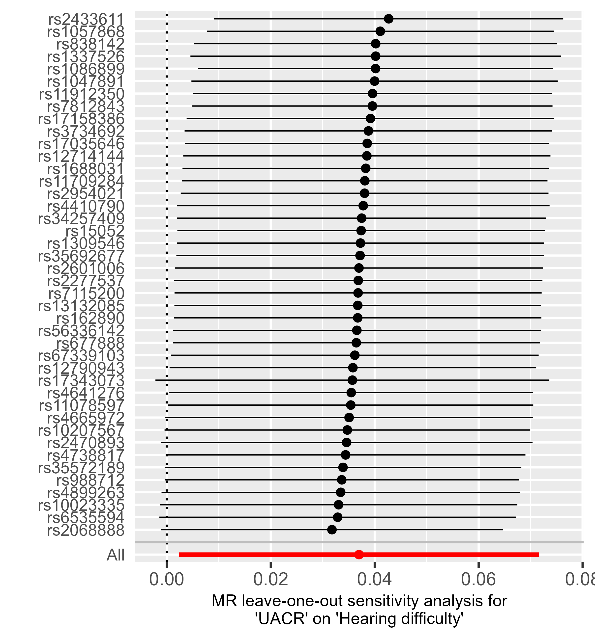


Within each panel, the black points represent the causal estimate of the association between a specific metabolite and epilepsy after discarding each SNP in turn. Red points represent the pooled IVW estimates. Horizontal lines denote 95% confidence intervals. MR, Mendelian randomization; HD, hearing difficulty; SNP, single nucleotide polymorphism; IVW, inverse variance-weighted.

**Figure S2 Funnel plot of the MR analysis for causal association of renal function with hearing loss.**

A) eGFR_cre; B) eGFR_cys; C) UACR; D) Urate. X-axis presented the causal estimates and Y axis presented the inverse SE. The dots indicated each SNP, and the line indicated the overall estimate using random-effect IVW method. MR, Mendelian Randomization; SE, Standard Error; SNP, Single Nucleotide Polymorphism; IVW, Inverse Variance-Weighted.
